# Supplementary material for: Gene expression profiles of Japanese precious coral Corallium japonicum during gametogenesis
Source: PeerJ. 2024 Apr 16;12:e17182. doi: 10.7717/peerj.17182 (PMC11027906; doi:10.7717/peerj.17182)
Supplement: Supplemental Information 1 [file peerj-12-17182-s001.pdf]

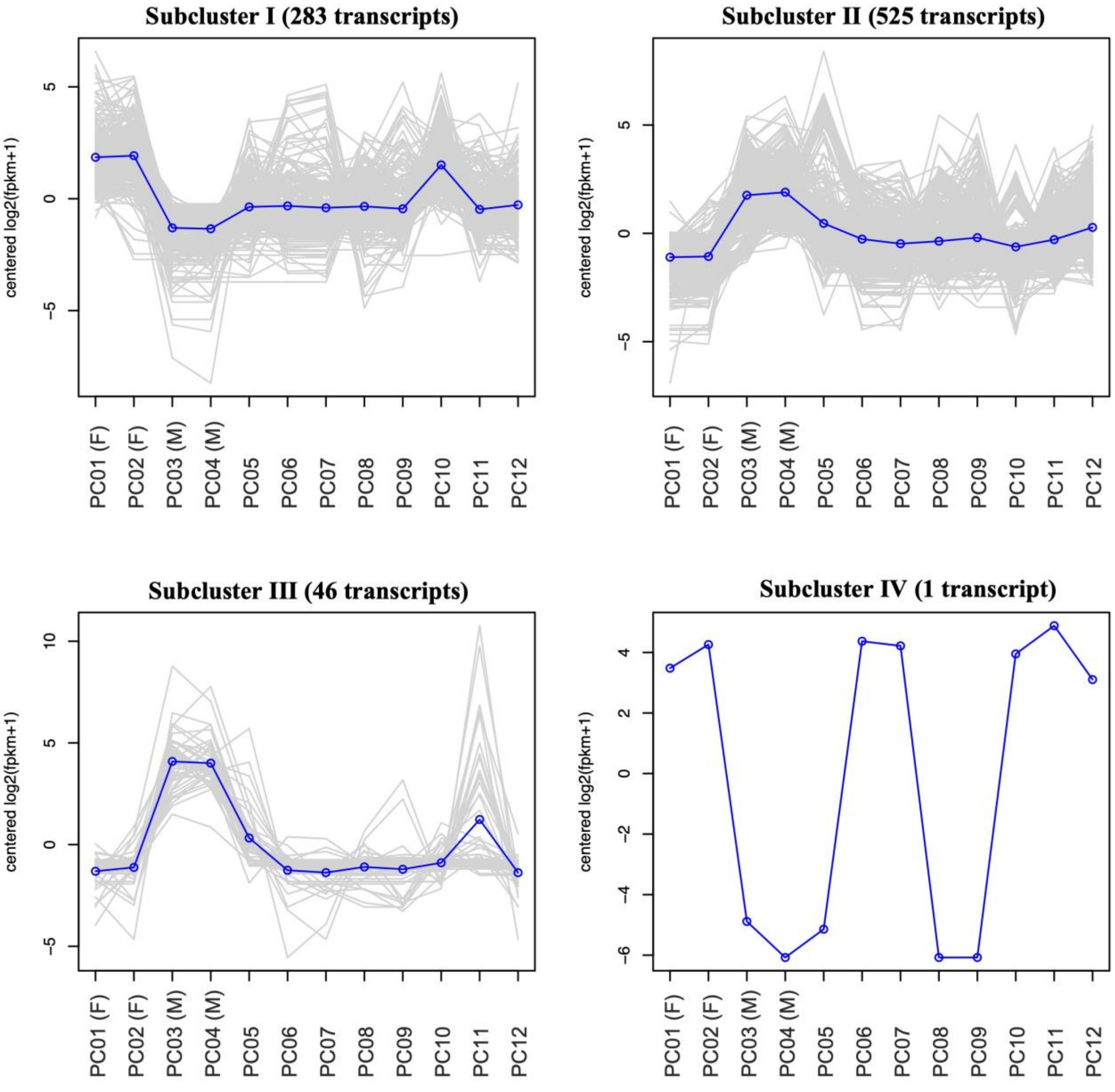
Subcluster I (283 transcripts)
centered log2(fpkm+1)
5
0
-5
PC01 (F)
PC02 (F)
PC03 (M)
PC04 (M)
PC05
PC06
PC07
PC08
PC09
PC10
PC11
PC12
Subcluster II (525 transcripts)
centered log2(fpkm+1)
5
0
-5
PC01 (F)
PC02 (F)
PC03 (M)
PC04 (M)
PC05
PC06
PC07
PC08
PC09
PC10
PC11
PC12
Subcluster III (46 transcripts)
centered log2(fpkm+1)
10
5
0
-5
PC01 (F)
PC02 (F)
PC03 (M)
PC04 (M)
PC05
PC06
PC07
PC08
PC09
PC10
PC11
PC12
Subcluster IV (1 transcript)
centered log2(fpkm+1)
4
2
0
-2
-4
-6
PC01 (F)
PC02 (F)
PC03 (M)
PC04 (M)
PC05
PC06
PC07
PC08
PC09
PC10
PC11
PC12
