# Supplementary figures and images for: Gene expression profiles of Japanese precious coral Corallium japonicum during gametogenesis
Source: PeerJ. 2024 Apr 16;12:e17182. doi: 10.7717/peerj.17182 (PMC11027906; doi:10.7717/peerj.17182)

A

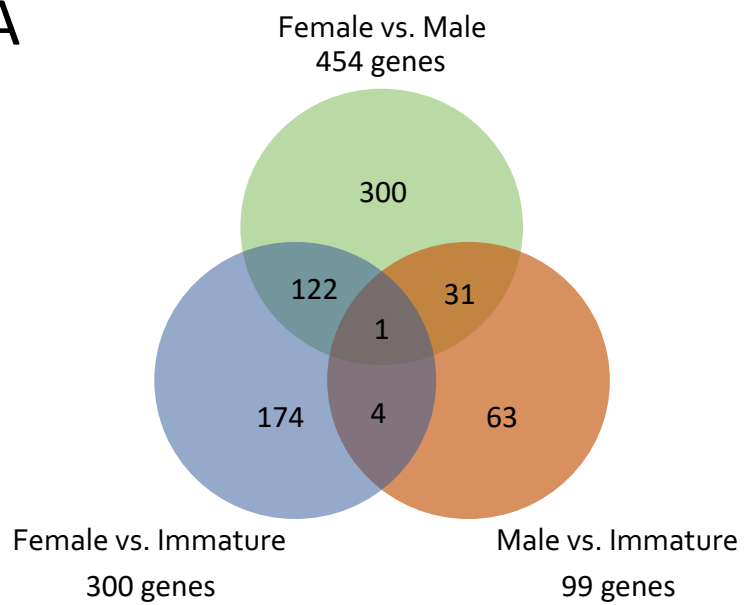

B

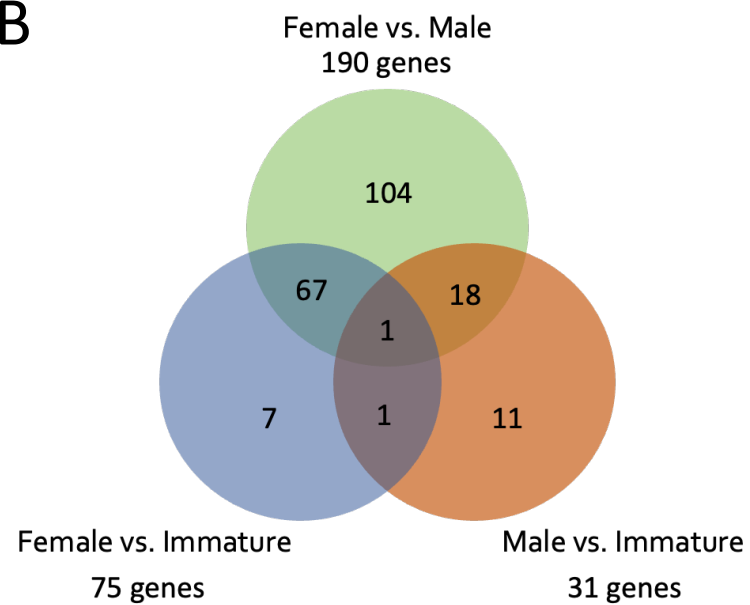

Supplement: Supplemental Information 2 [file peerj-12-17182-s002.pdf]

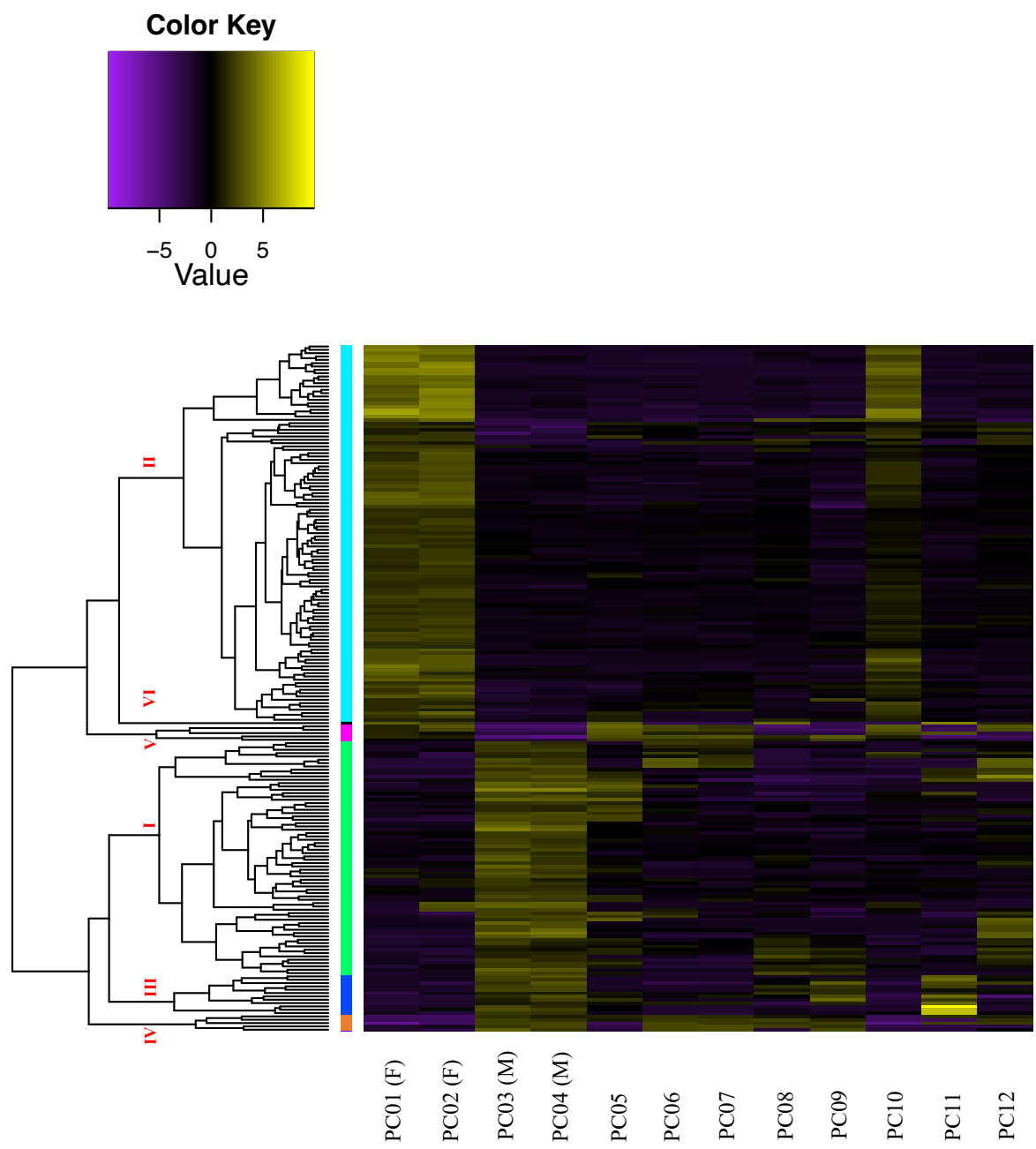

Supplement: Supplemental Information 3 — Cluster numbers are shown in red font, Roman numerals. Genes belonging to subcluster I are grouped in light green vertical bars, while genes under subcluster II are grouped in light blue vertical bars. [file peerj-12-17182-s003.pdf]
