# Supplementary material for: Gene expression profiles of Japanese precious coral Corallium japonicum during gametogenesis
Source: PeerJ. 2024 Apr 16;12:e17182. doi: 10.7717/peerj.17182 (PMC11027906; doi:10.7717/peerj.17182)
Supplement: Supplemental Information 4 [file peerj-12-17182-s004.docx]

**Supplemental Table 1.** RNA concentration and RIN values of the 12 *Corallium japoncium* samples.

| **Sample ID** | **Concentration (ng/uL)** | **RIN** |
| --- | --- | --- |
| PC01 | 27.0 | 8.3 |
| PC02 | 10.0 | N/A |
| PC03 | 13.0 | 7.0 |
| PC04 | 41.0 | 7.8 |
| PC05 | 31.0 | 8.4 |
| PC06 | 40.0 | 7.5 |
| PC07 | 25.0 | 7.2 |
| PC08 | 44.0 | 8.7 |
| PC09 | 16.0 | 7.6 |
| PC10 | 7.0 | N/A |
| PC11 | 12.0 | 4.8 |
| PC12 | 40.0 | 7.4 |
|  |  |  |
|  |  |  |
| min | 7.0 | 4.8 |
| max | 44.0 | 8.7 |
