# Supplementary material for: Gene expression profiles of Japanese precious coral Corallium japonicum during gametogenesis
Source: PeerJ. 2024 Apr 16;12:e17182. doi: 10.7717/peerj.17182 (PMC11027906; doi:10.7717/peerj.17182)
Supplement: Supplemental Information 5 [file peerj-12-17182-s005.docx]

**Supplemental Table 2A.** Result of the similarity search for DNA sequences (blastx) between female de novo assembled *C. japonicum* transcripts and contigs mapped with *C. rubrum*. Data only shows the most significant hits (<0.001).

| **No.** | **saccver** | **qaccver** | **pident** | **length** | **mismatch** | **gapopen** | **qstart** | **qend** | **sstart** | **send** | **evalue** | **bitscore** |
| --- | --- | --- | --- | --- | --- | --- | --- | --- | --- | --- | --- | --- |
| 1 | TRINITY_DN22915_c0_g1_i1.p1 | Contig_245 | 99.1 | 112 | 1 | 0 | 540 | 205 | 1 | 112 | 1.63E-74 | 220 |
| 2 | TRINITY_DN4236_c0_g1_i1.p1 | Contig_3066 | 99.1 | 218 | 2 | 0 | 3216 | 2563 | 1 | 218 | 5.16E-148 | 442 |
| 3 | TRINITY_DN7272_c0_g1_i28.p1 | Contig_5342 | 77.9 | 271 | 59 | 1 | 1082 | 273 | 7 | 277 | 5.13E-149 | 422 |
| 4 | TRINITY_DN364_c0_g1_i2.p1 | Contig_6045 | 95.1 | 122 | 6 | 0 | 596 | 231 | 5 | 126 | 8.18E-62 | 192 |
| 5 | TRINITY_DN100339_c0_g1_i6.p1 | Contig_6126 | 98.8 | 247 | 3 | 0 | 65 | 805 | 1 | 247 | 3.06E-100 | 296 |
| 6 | TRINITY_DN8312_c0_g1_i1.p1 | Contig_6599 | 96.4 | 166 | 6 | 0 | 39 | 536 | 2 | 167 | 3.09E-116 | 330 |
| 7 | TRINITY_DN47850_c0_g1_i1.p1 | Contig_6914 | 97.8 | 179 | 4 | 0 | 937 | 401 | 1 | 179 | 2.55E-123 | 350 |
| 8 | TRINITY_DN8537_c0_g1_i2.p1 | Contig_7128 | 97.3 | 525 | 14 | 0 | 1804 | 230 | 1 | 525 | 0 | 1034 |
| 9 | TRINITY_DN726_c0_g1_i1.p1 | Contig_7184 | 99 | 402 | 4 | 0 | 74 | 1279 | 1 | 402 | 7.38E-282 | 773 |
| 10 | TRINITY_DN33062_c0_g1_i1.p1 | Contig_7912 | 84.3 | 669 | 23 | 4 | 2022 | 208 | 13 | 663 | 0 | 997 |
| 11 | TRINITY_DN36259_c0_g1_i1.p1 | Contig_8004 | 97.3 | 789 | 21 | 0 | 100 | 2466 | 1 | 789 | 0 | 1484 |
| 12 | TRINITY_DN8472_c0_g1_i3.p1 | Contig_8252 | 97.2 | 789 | 21 | 1 | 3366 | 5732 | 1 | 788 | 0 | 1469 |
| 13 | TRINITY_DN26534_c0_g1_i1.p1 | Contig_8355 | 98.8 | 330 | 4 | 0 | 1032 | 43 | 1 | 330 | 1.13E-209 | 577 |
| 14 | TRINITY_DN21245_c0_g1_i2.p2 | Contig_8627 | 100 | 119 | 0 | 0 | 84 | 440 | 4 | 122 | 3.79E-74 | 226 |
| 15 | TRINITY_DN23865_c0_g1_i1.p1 | Contig_9007 | 99.6 | 253 | 1 | 0 | 1402 | 644 | 1 | 253 | 2.44E-171 | 485 |
| 16 | TRINITY_DN59854_c0_g2_i1.p1 | Contig_9434 | 98.6 | 74 | 1 | 0 | 1994 | 1773 | 1 | 74 | 3.54E-27 | 107 |
| 17 | TRINITY_DN2425_c0_g2_i1.p1 | Contig_9467 | 97.6 | 572 | 13 | 1 | 212 | 1927 | 1 | 571 | 0 | 1089 |
| 18 | TRINITY_DN16152_c0_g1_i2.p1 | Contig_10463 | 98.1 | 265 | 5 | 0 | 256 | 1050 | 212 | 476 | 1.44E-183 | 523 |
| 19 | TRINITY_DN6969_c1_g1_i2.p1 | Contig_10501 | 97.4 | 267 | 7 | 0 | 1154 | 354 | 73 | 339 | 4.71E-200 | 566 |
| 20 | TRINITY_DN144992_c0_g1_i4.p1 | Contig_11327 | 83.7 | 43 | 7 | 0 | 141 | 269 | 4 | 46 | 5.01E-16 | 73.2 |
| 21 | TRINITY_DN1115_c0_g1_i4.p1 | Contig_11661 | 92.2 | 115 | 8 | 1 | 23 | 367 | 31 | 144 | 6.78E-72 | 217 |
| 22 | TRINITY_DN25887_c0_g2_i1.p1 | Contig_11740 | 76.4 | 106 | 25 | 0 | 48 | 365 | 6 | 111 | 7.37E-45 | 150 |
| 23 | TRINITY_DN23830_c0_g1_i4.p1 | Contig_12847 | 94.3 | 122 | 7 | 0 | 140 | 505 | 1 | 122 | 1.90E-77 | 228 |
| 24 | TRINITY_DN46912_c0_g1_i1.p1 | Contig_13041 | 80.8 | 26 | 5 | 0 | 11 | 88 | 3 | 28 | 7.36E-11 | 54.7 |
| 25 | TRINITY_DN43579_c1_g1_i1.p1 | Contig_13779 | 80 | 30 | 6 | 0 | 1 | 90 | 14 | 43 | 7.53E-10 | 54.7 |
| 26 | TRINITY_DN5447_c0_g1_i3.p1 | Contig_16225 | 94.8 | 484 | 25 | 0 | 1629 | 178 | 1 | 484 | 4.78E-281 | 787 |
| 27 | TRINITY_DN9942_c0_g1_i3.p1 | Contig_16588 | 88.9 | 297 | 12 | 1 | 2942 | 3832 | 5 | 280 | 1.93E-188 | 554 |
| 28 | TRINITY_DN4240_c1_g1_i2.p1 | Contig_17081 | 95 | 302 | 14 | 1 | 18 | 920 | 1 | 302 | 2.04E-205 | 567 |
| 29 | TRINITY_DN5482_c1_g1_i5.p1 | Contig_17286 | 91.4 | 163 | 14 | 0 | 52 | 540 | 1 | 163 | 5.03E-97 | 292 |
| 30 | TRINITY_DN992_c0_g1_i3.p1 | Contig_17293 | 98.6 | 366 | 5 | 0 | 185 | 1282 | 1 | 366 | 1.01E-256 | 711 |
| 31 | TRINITY_DN9953_c0_g1_i1.p1 | Contig_17323 | 97.3 | 183 | 5 | 0 | 1043 | 495 | 1 | 183 | 4.39E-122 | 350 |
| 32 | TRINITY_DN10043_c0_g1_i3.p1 | Contig_17805 | 99.4 | 316 | 2 | 0 | 1448 | 501 | 2 | 317 | 2.51E-230 | 634 |
| 33 | TRINITY_DN9140_c0_g1_i1.p1 | Contig_18078 | 99.1 | 528 | 5 | 0 | 147 | 1730 | 1 | 528 | 0 | 1084 |
| 34 | TRINITY_DN1441_c0_g1_i1.p1 | Contig_18133 | 98.1 | 958 | 17 | 1 | 189 | 3059 | 1 | 958 | 0 | 1750 |
| 35 | TRINITY_DN18657_c0_g5_i1.p1 | Contig_20776 | 99.5 | 205 | 1 | 0 | 47 | 661 | 1 | 205 | 3.21E-155 | 432 |
| 36 | TRINITY_DN2634_c0_g1_i2.p1 | Contig_21128 | 63.9 | 61 | 16 | 3 | 563 | 399 | 85 | 145 | 3.11E-05 | 42.4 |
| 37 | TRINITY_DN12964_c0_g1_i37.p1 | Contig_22444 | 99.2 | 122 | 1 | 0 | 907 | 542 | 1 | 122 | 2.09E-83 | 249 |
| 38 | TRINITY_DN176_c0_g1_i1.p1 | Contig_23206 | 89.8 | 352 | 35 | 1 | 1212 | 160 | 5 | 356 | 1.88E-158 | 451 |
| 39 | TRINITY_DN7752_c0_g1_i2.p1 | Contig_23368 | 96.8 | 154 | 5 | 0 | 955 | 494 | 1 | 154 | 3.41E-106 | 307 |
| 40 | TRINITY_DN3907_c0_g1_i10.p1 | Contig_24420 | 98.9 | 186 | 2 | 0 | 705 | 148 | 3 | 188 | 2.72E-133 | 374 |
| 41 | TRINITY_DN15375_c0_g2_i1.p1 | Contig_25058 | 99.3 | 274 | 2 | 0 | 144 | 965 | 1 | 274 | 7.47E-196 | 543 |
| 42 | TRINITY_DN13336_c0_g1_i4.p1 | Contig_25108 | 98.9 | 358 | 4 | 0 | 1305 | 232 | 1 | 358 | 2.30E-271 | 738 |
| 43 | TRINITY_DN14963_c0_g1_i22.p1 | Contig_28281 | 93.1 | 304 | 19 | 1 | 176 | 1087 | 1 | 302 | 2.65E-189 | 546 |
| 44 | TRINITY_DN7863_c0_g1_i7.p1 | Contig_28513 | 96.8 | 252 | 8 | 0 | 1179 | 424 | 1 | 252 | 6.29E-165 | 464 |
| 45 | TRINITY_DN9054_c0_g2_i1.p1 | Contig_28861 | 51.4 | 547 | 241 | 6 | 117 | 1712 | 21 | 557 | 3.97E-200 | 577 |
| 46 | TRINITY_DN164369_c1_g1_i1.p1 | Contig_30329 | 54.5 | 530 | 186 | 5 | 2147 | 714 | 1 | 527 | 4.27E-165 | 513 |
| 47 | TRINITY_DN29716_c0_g1_i1.p1 | Contig_30333 | 97.3 | 258 | 4 | 2 | 76 | 846 | 1 | 256 | 1.60E-171 | 483 |
| 48 | TRINITY_DN10913_c0_g1_i15.p1 | Contig_30712 | 98 | 250 | 5 | 0 | 2969 | 2220 | 1 | 250 | 8.57E-173 | 507 |
| 49 | TRINITY_DN5786_c0_g1_i1.p1 | Contig_30994 | 99.3 | 301 | 2 | 0 | 264 | 1166 | 1 | 301 | 4.57E-219 | 605 |
| 50 | TRINITY_DN102349_c0_g2_i4.p1 | Contig_31564 | 50.9 | 517 | 233 | 11 | 2099 | 585 | 1 | 508 | 7.41E-161 | 474 |
| 51 | TRINITY_DN2711_c0_g1_i1.p1 | Contig_31624 | 96.6 | 238 | 8 | 0 | 902 | 189 | 1 | 238 | 2.74E-168 | 471 |
| 52 | TRINITY_DN51440_c0_g1_i1.p1 | Contig_31651 | 91.2 | 285 | 25 | 0 | 95 | 949 | 1 | 285 | 7.64E-134 | 384 |
| 53 | TRINITY_DN22304_c0_g1_i1.p1 | Contig_31725 | 98.3 | 708 | 12 | 0 | 2362 | 239 | 1 | 708 | 0 | 1408 |
| 54 | TRINITY_DN2892_c0_g1_i1.p1 | Contig_31809 | 68.7 | 233 | 8 | 1 | 1 | 699 | 53 | 220 | 9.34E-93 | 274 |
| 55 | TRINITY_DN32497_c0_g1_i2.p1 | Contig_31858 | 95.9 | 315 | 13 | 0 | 1038 | 94 | 1 | 315 | 1.94E-202 | 561 |
| 56 | TRINITY_DN7259_c0_g1_i1.p1 | Contig_31862 | 100 | 296 | 0 | 0 | 1435 | 548 | 1 | 296 | 4.51E-216 | 598 |
| 57 | TRINITY_DN78650_c2_g1_i1.p1 | Contig_32119 | 72.7 | 22 | 6 | 0 | 390 | 455 | 82 | 103 | 1.97E-04 | 38.5 |
| 58 | TRINITY_DN37579_c1_g1_i1.p1 | Contig_32496 | 97.5 | 360 | 9 | 0 | 82 | 1161 | 1 | 360 | 2.53E-255 | 697 |
| 59 | TRINITY_DN4069_c0_g1_i4.p1 | Contig_32685 | 99.4 | 314 | 2 | 0 | 1295 | 354 | 1 | 314 | 2.48E-229 | 633 |
| 60 | TRINITY_DN9766_c0_g1_i1.p1 | Contig_32689 | 94.9 | 333 | 17 | 0 | 188 | 1186 | 7 | 339 | 2.98E-121 | 357 |
| 61 | TRINITY_DN2412_c0_g1_i10.p1 | Contig_33300 | 81.8 | 384 | 69 | 1 | 1411 | 260 | 1 | 383 | 1.35E-221 | 615 |
| 62 | TRINITY_DN12605_c0_g1_i2.p1 | Contig_33986 | 94.4 | 161 | 9 | 0 | 25 | 507 | 27 | 187 | 3.73E-102 | 292 |
| 63 | TRINITY_DN3478_c0_g1_i1.p1 | Contig_35869 | 98.7 | 456 | 4 | 1 | 195 | 1562 | 1 | 454 | 7.36e-310 | 845 |
| 64 | TRINITY_DN37965_c1_g1_i5.p1 | Contig_35910 | 95 | 424 | 21 | 0 | 79 | 1350 | 1 | 424 | 2.44E-227 | 656 |
| 65 | TRINITY_DN6819_c0_g1_i1.p1 | Contig_36030 | 99 | 286 | 3 | 0 | 128 | 985 | 1 | 286 | 5.98E-208 | 574 |
| 66 | TRINITY_DN16152_c0_g1_i2.p1 | Contig_36148 | 77.9 | 471 | 98 | 4 | 1123 | 2529 | 10 | 476 | 8.67E-230 | 686 |
| 67 | TRINITY_DN9169_c0_g1_i2.p1 | Contig_36298 | 92.9 | 253 | 10 | 2 | 129 | 887 | 1 | 245 | 2.45E-136 | 388 |
| 68 | TRINITY_DN2634_c0_g1_i2.p1 | Contig_36323 | 64.3 | 129 | 34 | 4 | 3 | 371 | 23 | 145 | 1.95E-28 | 101 |
| 69 | TRINITY_DN3623_c1_g1_i2.p1 | Contig_37617 | 92.5 | 187 | 14 | 0 | 1588 | 1028 | 1 | 187 | 1.20E-99 | 301 |
| 70 | TRINITY_DN11740_c0_g1_i14.p1 | Contig_38830 | 97.2 | 176 | 4 | 1 | 1446 | 919 | 1 | 175 | 1.31E-103 | 325 |
| 71 | TRINITY_DN21245_c0_g1_i2.p1 | Contig_39917 | 100 | 126 | 0 | 0 | 600 | 977 | 1 | 126 | 5.73E-79 | 247 |
| 72 | TRINITY_DN21245_c0_g1_i2.p2 | Contig_40080 | 100 | 98 | 0 | 0 | 643 | 350 | 1 | 98 | 8.17E-61 | 187 |
| 73 | TRINITY_DN8281_c0_g1_i1.p1 | Contig_40910 | 94.7 | 75 | 4 | 0 | 103 | 327 | 1 | 75 | 3.09E-34 | 123 |
| 74 | TRINITY_DN5745_c0_g1_i1.p1 | Contig_40947 | 97.7 | 133 | 2 | 1 | 68 | 466 | 12 | 143 | 3.58E-76 | 227 |
| 75 | TRINITY_DN176_c0_g1_i1.p1 | Contig_41494 | 72.7 | 143 | 28 | 2 | 757 | 329 | 193 | 324 | 1.03E-62 | 207 |
| 76 | TRINITY_DN11641_c1_g1_i14.p1 | Contig_41779 | 100 | 114 | 0 | 0 | 48 | 389 | 1 | 114 | 8.10E-77 | 226 |
| 77 | TRINITY_DN6454_c0_g1_i1.p1 | Contig_41918 | 100 | 146 | 0 | 0 | 45 | 482 | 1 | 146 | 9.13E-105 | 303 |
| 78 | TRINITY_DN1592_c4_g1_i1.p1 | Contig_41933 | 89.2 | 223 | 22 | 2 | 325 | 990 | 1 | 222 | 7.28E-134 | 383 |
| 79 | TRINITY_DN6986_c0_g1_i1.p1 | Contig_42362 | 98.2 | 451 | 8 | 0 | 1729 | 377 | 1 | 451 | 6.39E-305 | 835 |
| 80 | TRINITY_DN7778_c0_g1_i1.p1 | Contig_42743 | 97 | 607 | 16 | 1 | 46 | 1860 | 1 | 607 | 0 | 1096 |
| 81 | TRINITY_DN1887_c0_g1_i1.p1 | Contig_42960 | 96.6 | 232 | 8 | 0 | 887 | 192 | 1 | 232 | 1.43E-159 | 445 |
| 82 | TRINITY_DN5587_c0_g2_i1.p1 | Contig_43127 | 98.1 | 108 | 2 | 0 | 29 | 352 | 1 | 108 | 1.53E-62 | 189 |
| 83 | TRINITY_DN8282_c0_g2_i1.p1 | Contig_43155 | 99.5 | 196 | 1 | 0 | 132 | 719 | 1 | 196 | 4.67E-128 | 365 |
| 84 | TRINITY_DN6952_c0_g1_i1.p1 | Contig_43554 | 97.2 | 288 | 8 | 0 | 340 | 1203 | 1 | 288 | 2.72E-211 | 586 |
| 85 | TRINITY_DN4241_c0_g1_i1.p1 | Contig_43825 | 95.8 | 118 | 5 | 0 | 86 | 439 | 1 | 118 | 5.42E-88 | 253 |
| 86 | TRINITY_DN30869_c0_g2_i1.p1 | Contig_43923 | 97.1 | 971 | 27 | 1 | 3291 | 382 | 1 | 971 | 0 | 1779 |
| 87 | TRINITY_DN14509_c0_g1_i1.p1 | Contig_43962 | 95.3 | 408 | 18 | 1 | 195 | 1418 | 1 | 407 | 2.02E-273 | 747 |
| 88 | TRINITY_DN13525_c0_g1_i2.p1 | Contig_44079 | 97.5 | 197 | 4 | 1 | 1066 | 479 | 1 | 197 | 3.18E-88 | 265 |
| 89 | TRINITY_DN176_c0_g1_i1.p1 | Contig_44174 | 50.1 | 385 | 146 | 4 | 1437 | 292 | 1 | 342 | 1.23E-83 | 263 |
| 90 | TRINITY_DN18911_c1_g1_i1.p1 | Contig_44188 | 95.5 | 693 | 20 | 1 | 168 | 2246 | 1 | 682 | 0 | 1229 |
| 91 | TRINITY_DN4520_c0_g1_i2.p1 | Contig_44275 | 94.9 | 415 | 18 | 2 | 1189 | 2433 | 23 | 434 | 1.85E-266 | 750 |
| 92 | TRINITY_DN31999_c1_g1_i1.p1 | Contig_44356 | 99.1 | 329 | 3 | 0 | 196 | 1182 | 1 | 329 | 1.59E-222 | 620 |
| 93 | TRINITY_DN6317_c0_g1_i1.p1 | Contig_45343 | 97.4 | 268 | 7 | 0 | 1126 | 323 | 1 | 268 | 3.17E-179 | 499 |
| 94 | TRINITY_DN24362_c0_g1_i7.p1 | Contig_45427 | 45.6 | 443 | 227 | 3 | 185 | 1492 | 12 | 447 | 5.49E-136 | 403 |
| 95 | TRINITY_DN3165_c0_g2_i1.p1 | Contig_45710 | 94.9 | 525 | 27 | 0 | 110 | 1684 | 1 | 525 | 0 | 896 |
| 96 | TRINITY_DN176_c0_g1_i1.p1 | Contig_46256 | 89.7 | 203 | 21 | 0 | 609 | 1 | 55 | 257 | 1.05E-60 | 199 |
| 97 | TRINITY_DN10744_c0_g1_i3.p1 | Contig_46897 | 99.2 | 130 | 1 | 0 | 1479 | 1090 | 1 | 130 | 2.55E-80 | 247 |
| 98 | TRINITY_DN176_c0_g1_i1.p1 | Contig_47142 | 78.9 | 57 | 12 | 0 | 149 | 319 | 1 | 57 | 6.59E-26 | 103 |
| 99 | TRINITY_DN176_c0_g1_i1.p1 | Contig_47550 | 93.7 | 253 | 14 | 1 | 119 | 871 | 1 | 253 | 6.30E-97 | 291 |
| 100 | TRINITY_DN2217_c0_g1_i1.p1 | Contig_47962 | 96.1 | 152 | 6 | 0 | 578 | 1033 | 140 | 291 | 9.16E-99 | 295 |
|  |  |  |  |  |  |  |  |  |  |  |  |  |
|  |  |  |  |  |  |  |  |  |  |  |  |  |
|  |  | Min | 45.6 | 22 |  |  |  |  |  |  | 0.E+00 | 38.5 |
|  |  | Max | 100 | 971 |  |  |  |  |  |  | 2.E-04 | 1779 |
|  |  | Average | 91.58 | 296 |  |  |  |  |  |  | 2.E-06 | 508.57 |
|  |  | SD | 12.42 | 196 |  |  |  |  |  |  | 2.E-05 | 357.61 |
