# Supplementary material for: Gene expression profiles of Japanese precious coral Corallium japonicum during gametogenesis
Source: PeerJ. 2024 Apr 16;12:e17182. doi: 10.7717/peerj.17182 (PMC11027906; doi:10.7717/peerj.17182)
Supplement: Supplemental Information 6 [file peerj-12-17182-s006.docx]

**Supplemental Table 2B.** Result of the similarity search for DNA sequences (blastx) between male de novo assembled *C. japonicum* transcripts and contigs mapped with *C. rubrum*. Data only shows the most significant hits (<0.001).

| **Contog No.** | **De novo assembled transcripts** | **Reference-based transcripts** | **pident** | **length** | **mismatch** | **gapopen** | **qstart** | **qend** | **sstart** | **send** | **evalue** | **bitscore** |
| --- | --- | --- | --- | --- | --- | --- | --- | --- | --- | --- | --- | --- |
| 1 | TRINITY_DN51752_c0_g1_i1.p1 | Contig_439 | 98.5 | 269 | 4 | 0 | 59 | 865 | 1 | 269 | 8.22E-138 | 391 |
| 2 | TRINITY_DN20730_c0_g1_i2.p1 | Contig_1479 | 85.7 | 168 | 24 | 0 | 467 | 970 | 2 | 169 | 7.11E-94 | 289 |
| 3 | TRINITY_DN61756_c0_g1_i3.p2 | Contig_1488 | 81.6 | 49 | 9 | 0 | 539 | 685 | 177 | 225 | 2.25E-18 | 81.6 |
| 4 | TRINITY_DN12609_c0_g2_i1.p1 | Contig_2653 | 58.7 | 271 | 108 | 3 | 220 | 1029 | 4 | 271 | 2.42E-106 | 317 |
| 5 | TRINITY_DN273_c0_g1_i2.p2 | Contig_2678 | 80.5 | 82 | 14 | 1 | 880 | 641 | 1 | 82 | 1.58E-38 | 132 |
| 6 | TRINITY_DN27707_c1_g1_i8.p1 | Contig_4700 | 92.9 | 28 | 2 | 0 | 240 | 323 | 1 | 28 | 2.26E-12 | 60.1 |
| 7 | TRINITY_DN1961_c0_g1_i3.p1 | Contig_6053 | 100 | 109 | 0 | 0 | 703 | 377 | 1 | 109 | 9.10E-71 | 212 |
| 8 | TRINITY_DN575_c1_g1_i18.p1 | Contig_6884 | 98.3 | 419 | 4 | 2 | 1714 | 458 | 1 | 416 | 1.80E-304 | 846 |
| 9 | TRINITY_DN37514_c1_g1_i10.p1 | Contig_7033 | 82.6 | 23 | 4 | 0 | 70 | 2 | 46 | 68 | 9.24E-06 | 43.1 |
| 10 | TRINITY_DN653_c0_g1_i1.p1 | Contig_9108 | 97.4 | 938 | 24 | 0 | 68 | 2881 | 1 | 938 | 0 | 1790 |
| 11 | TRINITY_DN64_c0_g1_i13.p1 | Contig_10215 | 80.1 | 136 | 27 | 0 | 1254 | 847 | 60 | 195 | 1.19E-73 | 234 |
| 12 | TRINITY_DN10772_c0_g1_i6.p1 | Contig_10459 | 60.9 | 266 | 104 | 0 | 51 | 848 | 11 | 276 | 2.25E-96 | 285 |
| 13 | TRINITY_DN9935_c0_g1_i3.p1 | Contig_10474 | 64.2 | 81 | 25 | 2 | 100 | 336 | 19 | 97 | 8.35E-26 | 94.7 |
| 14 | TRINITY_DN13206_c0_g1_i4.p1 | Contig_11067 | 64 | 422 | 146 | 4 | 1308 | 2561 | 49 | 468 | 6.52E-182 | 541 |
| 15 | TRINITY_DN38_c0_g1_i1.p2 | Contig_11243 | 88.7 | 62 | 7 | 0 | 1315 | 1130 | 33 | 94 | 4.94E-28 | 109 |
| 16 | TRINITY_DN5237_c0_g1_i4.p1 | Contig_11265 | 51.8 | 363 | 151 | 6 | 294 | 1358 | 1 | 347 | 1.17E-111 | 338 |
| 17 | TRINITY_DN562_c0_g1_i24.p1 | Contig_11323 | 71.6 | 116 | 25 | 3 | 325 | 2 | 1 | 116 | 4.44E-32 | 111 |
| 18 | TRINITY_DN5610_c0_g1_i6.p1 | Contig_12633 | 86.9 | 329 | 11 | 2 | 1365 | 469 | 1 | 327 | 1.06E-184 | 524 |
| 19 | TRINITY_DN13284_c0_g1_i2.p1 | Contig_12697 | 90.5 | 190 | 18 | 0 | 159 | 728 | 1 | 190 | 2.23E-116 | 333 |
| 20 | TRINITY_DN70217_c0_g1_i7.p1 | Contig_15917 | 80.8 | 125 | 24 | 0 | 13 | 387 | 1 | 125 | 1.44E-67 | 199 |
| 21 | TRINITY_DN4584_c12_g1_i1.p1 | Contig_16233 | 97 | 236 | 7 | 0 | 445 | 1152 | 1 | 236 | 2.73E-116 | 349 |
| 22 | TRINITY_DN2208_c0_g1_i3.p1 | Contig_17660 | 100 | 216 | 0 | 0 | 236 | 883 | 1 | 216 | 1.46E-121 | 350 |
| 23 | TRINITY_DN1210_c0_g1_i1.p1 | Contig_18084 | 93.4 | 61 | 4 | 0 | 3 | 185 | 458 | 518 | 3.50E-31 | 117 |
| 24 | TRINITY_DN3919_c0_g2_i1.p1 | Contig_19350 | 88.2 | 161 | 19 | 0 | 759 | 1241 | 41 | 201 | 2.80E-100 | 301 |
| 25 | TRINITY_DN69_c0_g1_i1.p1 | Contig_19956 | 60.2 | 699 | 231 | 8 | 2318 | 231 | 44 | 698 | 4.32E-295 | 825 |
| 26 | TRINITY_DN3398_c0_g1_i38.p1 | Contig_20610 | 81.1 | 180 | 33 | 1 | 590 | 1129 | 52 | 230 | 2.43E-112 | 327 |
| 27 | TRINITY_DN96_c0_g1_i4.p1 | Contig_21856 | 78.6 | 103 | 20 | 1 | 29 | 331 | 89 | 191 | 3.42E-46 | 149 |
| 28 | TRINITY_DN4339_c0_g1_i1.p1 | Contig_22776 | 97.7 | 350 | 8 | 0 | 275 | 1324 | 1 | 350 | 7.19E-200 | 560 |
| 29 | TRINITY_DN5639_c0_g1_i2.p2 | Contig_24002 | 73.8 | 168 | 41 | 2 | 15 | 518 | 2 | 166 | 7.38E-76 | 224 |
| 30 | TRINITY_DN1974_c0_g1_i10.p1 | Contig_26188 | 94.7 | 170 | 8 | 1 | 253 | 762 | 1 | 169 | 1.82E-87 | 270 |
| 31 | TRINITY_DN1889_c1_g1_i5.p1 | Contig_26278 | 98.1 | 207 | 4 | 0 | 1370 | 750 | 52 | 258 | 1.52E-143 | 419 |
| 32 | TRINITY_DN5139_c0_g1_i6.p1 | Contig_26751 | 52.9 | 223 | 102 | 3 | 771 | 1436 | 96 | 316 | 7.19E-71 | 230 |
| 33 | TRINITY_DN19958_c1_g1_i21.p1 | Contig_26858 | 93.8 | 48 | 3 | 0 | 2 | 145 | 61 | 108 | 4.16E-25 | 96.3 |
| 34 | TRINITY_DN4944_c0_g1_i1.p1 | Contig_28162 | 94.6 | 242 | 13 | 0 | 947 | 1672 | 1 | 242 | 1.05E-140 | 426 |
| 35 | TRINITY_DN1667_c0_g1_i8.p1 | Contig_28440 | 40.8 | 934 | 511 | 17 | 331 | 3039 | 7 | 929 | 8.82E-229 | 685 |
| 36 | TRINITY_DN2677_c0_g1_i9.p1 | Contig_28909 | 85.2 | 243 | 34 | 1 | 1 | 729 | 77 | 317 | 7.55E-153 | 429 |
| 37 | TRINITY_DN3398_c0_g1_i38.p1 | Contig_29184 | 81.1 | 175 | 32 | 1 | 522 | 1 | 52 | 226 | 9.85E-108 | 309 |
| 38 | TRINITY_DN2686_c0_g1_i5.p1 | Contig_29953 | 60.8 | 627 | 228 | 9 | 1680 | 3536 | 72 | 688 | 1.74E-273 | 787 |
| 39 | TRINITY_DN69_c0_g1_i1.p1 | Contig_29957 | 81.7 | 240 | 29 | 3 | 128 | 814 | 463 | 698 | 1.12E-131 | 394 |
| 40 | TRINITY_DN9135_c0_g1_i2.p1 | Contig_30539 | 83.8 | 160 | 25 | 1 | 467 | 943 | 1 | 160 | 6.13E-102 | 298 |
| 41 | TRINITY_DN3536_c0_g1_i1.p1 | Contig_30846 | 72.9 | 170 | 42 | 3 | 1313 | 813 | 5 | 173 | 2.54E-69 | 219 |
| 42 | TRINITY_DN42934_c0_g1_i5.p2 | Contig_32188 | 85 | 100 | 12 | 1 | 413 | 123 | 1 | 100 | 4.17E-68 | 202 |
| 43 | TRINITY_DN50692_c0_g1_i3.p1 | Contig_32851 | 67.7 | 31 | 10 | 0 | 323 | 231 | 84 | 114 | 1.13E-06 | 45.4 |
| 44 | TRINITY_DN20730_c0_g1_i2.p1 | Contig_33186 | 86.3 | 168 | 23 | 0 | 986 | 483 | 2 | 169 | 2.18E-98 | 293 |
| 45 | TRINITY_DN120_c0_g1_i1.p1 | Contig_34328 | 76.4 | 157 | 37 | 0 | 365 | 835 | 18 | 174 | 1.07E-76 | 231 |
| 46 | TRINITY_DN1961_c0_g1_i3.p1 | Contig_35140 | 100 | 21 | 0 | 0 | 67 | 129 | 89 | 109 | 2.38E-06 | 43.5 |
| 47 | TRINITY_DN6823_c2_g1_i2.p1 | Contig_36211 | 57.1 | 105 | 43 | 1 | 607 | 921 | 48 | 150 | 1.53E-28 | 118 |
| 48 | TRINITY_DN4944_c0_g1_i1.p1 | Contig_36241 | 98.3 | 117 | 2 | 0 | 2112 | 2462 | 126 | 242 | 2.49E-68 | 228 |
| 49 | TRINITY_DN11286_c0_g1_i6.p4 | Contig_36626 | 85 | 40 | 6 | 0 | 443 | 562 | 79 | 118 | 1.48E-12 | 62.4 |
| 50 | TRINITY_DN15023_c0_g1_i9.p1 | Contig_36694 | 91.7 | 156 | 13 | 0 | 196 | 663 | 1 | 156 | 1.74E-103 | 299 |
| 51 | TRINITY_DN25641_c0_g1_i2.p1 | Contig_37440 | 93.1 | 160 | 11 | 0 | 848 | 1327 | 1 | 160 | 2.18E-101 | 312 |
| 52 | TRINITY_DN2488_c0_g1_i6.p1 | Contig_37568 | 79.7 | 158 | 32 | 0 | 133 | 606 | 1 | 158 | 5.70E-85 | 254 |
| 53 | TRINITY_DN8382_c0_g1_i2.p1 | Contig_38567 | 88.4 | 146 | 17 | 0 | 1 | 438 | 80 | 225 | 9.26E-92 | 266 |
| 54 | TRINITY_DN13284_c0_g1_i2.p1 | Contig_40520 | 90.8 | 153 | 14 | 0 | 279 | 737 | 15 | 167 | 1.13E-93 | 275 |
| 55 | TRINITY_DN7091_c0_g1_i1.p1 | Contig_41275 | 79.4 | 199 | 38 | 1 | 1523 | 936 | 1 | 199 | 1.35E-98 | 297 |
| 56 | TRINITY_DN9230_c0_g1_i32.p1 | Contig_41428 | 87.5 | 40 | 5 | 0 | 1 | 120 | 3 | 42 | 1.01E-19 | 81.3 |
| 57 | TRINITY_DN19633_c0_g1_i3.p1 | Contig_41890 | 79.1 | 91 | 18 | 1 | 313 | 44 | 4 | 94 | 3.80E-43 | 137 |
| 58 | TRINITY_DN17811_c0_g1_i9.p1 | Contig_43036 | 63.6 | 33 | 12 | 0 | 62 | 160 | 87 | 119 | 4.41E-07 | 46.6 |
| 59 | TRINITY_DN46728_c0_g1_i6.p1 | Contig_43354 | 90 | 90 | 9 | 0 | 189 | 458 | 46 | 135 | 3.08E-56 | 172 |
| 60 | TRINITY_DN2074_c0_g1_i1.p1 | Contig_44720 | 97.2 | 253 | 7 | 0 | 1052 | 294 | 1 | 253 | 5.82E-165 | 462 |
| 61 | TRINITY_DN4371_c0_g2_i1.p1 | Contig_46439 | 99.1 | 433 | 4 | 0 | 4522 | 3224 | 2 | 434 | 2.87E-294 | 841 |
| 62 | TRINITY_DN4229_c0_g1_i1.p1 | Contig_46637 | 94.6 | 56 | 3 | 0 | 2 | 169 | 59 | 114 | 8.08E-41 | 131 |
| 63 | TRINITY_DN69_c0_g1_i1.p1 | Contig_47813 | 86.5 | 654 | 78 | 4 | 1 | 1962 | 55 | 698 | 0 | 1160 |
|  |  |  |  |  |  |  |  |  |  |  |  |  |
|  |  |  |  |  |  |  |  |  |  |  |  |  |
|  |  | Min | 40.8 | 21 |  |  |  |  |  |  | 0.E+00 | 43.1 |
|  |  | Max | 100 | 938 |  |  |  |  |  |  | 9.E-06 | 1790 |
|  |  | Average | 82.58 | 213 |  |  |  |  |  |  | 2.E-07 | 327.81 |
|  |  | STDEV | 14.09 | 196 |  |  |  |  |  |  | 1.E-06 | 292.85 |
