# Supplementary material for: Gene expression profiles of Japanese precious coral Corallium japonicum during gametogenesis
Source: PeerJ. 2024 Apr 16;12:e17182. doi: 10.7717/peerj.17182 (PMC11027906; doi:10.7717/peerj.17182)
Supplement: Supplemental Information 8 [file peerj-12-17182-s008.docx]

**Supplemental Table 3B.** Result of the comparison between de novo assembled transcripts and reference-based assembled transcripts of male *C. japonicum*. Data only shows the most significant hits (<0.001).

| **saccver** | **qaccver** | **pident** | **length** | **mismatch** | **gapopen** | **qstart** | **qend** | **sstart** | **send** | **evalue** | **bitscore** |
| --- | --- | --- | --- | --- | --- | --- | --- | --- | --- | --- | --- |
| TRINITY_DN10744_c0_g1_i3 | Contig_46897 | 96.098 | 820 | 16 | 4 | 23 | 826 | 1670 | 851 | 0 | 1323 |
| TRINITY_DN10913_c0_g1_i15 | Contig_30712 | 95.347 | 1225 | 29 | 6 | 162 | 1378 | 2968 | 1764 | 0 | 1921 |
| TRINITY_DN11001_c0_g1_i3 | Contig_23206 | 96.897 | 419 | 12 | 1 | 1 | 418 | 963 | 545 | 0 | 701 |
|  | Contig_47550 | 93.317 | 419 | 27 | 1 | 1 | 418 | 374 | 792 | 6.89E-179 | 617 |
|  | Contig_44174 | 93.349 | 421 | 21 | 5 | 1 | 418 | 1173 | 757 | 2.48E-178 | 616 |
|  | Contig_46256 | 93.079 | 419 | 28 | 1 | 1 | 418 | 510 | 92 | 3.20E-177 | 612 |
|  | Contig_41494 | 90.476 | 378 | 27 | 9 | 1 | 371 | 1075 | 700 | 1.57E-140 | 490 |
| TRINITY_DN11001_c1_g1_i2 | Contig_23206 | 97.26 | 219 | 6 | 0 | 1 | 219 | 613 | 395 | 2.99E-105 | 372 |
|  | Contig_41494 | 94.091 | 220 | 12 | 1 | 1 | 219 | 690 | 471 | 1.41E-93 | 333 |
|  | Contig_44174 | 93.213 | 221 | 13 | 2 | 1 | 219 | 826 | 606 | 8.50E-91 | 324 |
|  | Contig_46256 | 97.5 | 160 | 4 | 0 | 1 | 160 | 160 | 1 | 8.68E-76 | 274 |
|  | Contig_47550 | 97.959 | 147 | 3 | 0 | 1 | 147 | 724 | 870 | 3.14E-70 | 255 |
| TRINITY_DN1115_c0_g1_i4 | Contig_11661 | 95.989 | 349 | 11 | 1 | 1 | 346 | 370 | 22 | 9.51E-163 | 564 |
| TRINITY_DN11289_c0_g1_i1 | Contig_36197 | 97.286 | 479 | 12 | 1 | 19 | 497 | 497 | 20 | 0 | 811 |
| TRINITY_DN11811_c0_g1_i1 | Contig_33300 | 81.064 | 940 | 146 | 25 | 456 | 1379 | 273 | 1196 | 0 | 721 |
| TRINITY_DN12185_c0_g1_i3 | Contig_38830 | 84.146 | 82 | 13 | 0 | 996 | 1077 | 1823 | 1904 | 1.06E-16 | 80.5 |
| TRINITY_DN13525_c0_g1_i2 | Contig_44079 | 96.435 | 1038 | 28 | 6 | 1 | 1033 | 131 | 1164 | 0 | 1703 |
| TRINITY_DN13555_c0_g1_i15 | Contig_11740 | 93.617 | 47 | 2 | 1 | 47 | 93 | 1174 | 1129 | 1.24E-13 | 69.4 |
| TRINITY_DN1363_c0_g1_i8 | Contig_46256 | 97.07 | 273 | 5 | 1 | 88 | 357 | 1090 | 818 | 1.35E-130 | 457 |
|  | Contig_47142 | 96.538 | 260 | 9 | 0 | 80 | 339 | 60 | 319 | 8.16E-123 | 431 |
|  | Contig_44174 | 97.6 | 250 | 6 | 0 | 108 | 357 | 1498 | 1249 | 2.94E-122 | 429 |
|  | Contig_41494 | 96.85 | 254 | 8 | 0 | 104 | 357 | 1400 | 1147 | 3.80E-121 | 425 |
|  | Contig_47550 | 89.837 | 246 | 19 | 2 | 112 | 357 | 62 | 301 | 1.11E-86 | 311 |
|  | Contig_23206 | 84.459 | 296 | 43 | 3 | 65 | 357 | 1331 | 1036 | 5.20E-80 | 289 |
| TRINITY_DN1441_c0_g1_i1 | Contig_18133 | 98.355 | 3222 | 44 | 5 | 2 | 3222 | 3247 | 34 | 0 | 5648 |
| TRINITY_DN14509_c0_g1_i1 | Contig_43962 | 96.97 | 1485 | 30 | 4 | 4 | 1478 | 1513 | 34 | 0 | 2479 |
| TRINITY_DN1531_c0_g1_i1 | Contig_16110 | 95.996 | 999 | 26 | 4 | 104 | 1096 | 1027 | 37 | 0 | 1611 |
|  | Contig_34698 | 96.89 | 611 | 10 | 2 | 381 | 990 | 663 | 61 | 0 | 1014 |
| TRINITY_DN15375_c0_g2_i1 | Contig_25058 | 97.398 | 1345 | 25 | 5 | 217 | 1555 | 1341 | 1 | 0 | 2281 |
| TRINITY_DN1592_c4_g1_i1 | Contig_41933 | 92.316 | 937 | 43 | 16 | 21 | 953 | 1235 | 324 | 0 | 1304 |
| TRINITY_DN16128_c1_g1_i6 | Contig_13779 | 84.224 | 393 | 58 | 3 | 1 | 391 | 224 | 614 | 3.30E-107 | 379 |
|  | Contig_13041 | 88.673 | 309 | 33 | 2 | 34 | 341 | 9 | 316 | 4.27E-106 | 375 |
| TRINITY_DN16152_c0_g1_i2 | Contig_36148 | 94.879 | 1445 | 58 | 6 | 1 | 1430 | 2509 | 3952 | 0 | 2244 |
|  | Contig_10463 | 96.103 | 1052 | 40 | 1 | 380 | 1430 | 1 | 1052 | 0 | 1714 |
|  | Contig_36148 | 95.218 | 711 | 20 | 1 | 1 | 697 | 3625 | 4335 | 0 | 1112 |
|  | Contig_10463 | 90.503 | 358 | 34 | 0 | 1 | 358 | 739 | 1096 | 2.68E-135 | 473 |
| TRINITY_DN1887_c0_g1_i1 | Contig_42960 | 97.596 | 915 | 16 | 3 | 159 | 1068 | 1005 | 92 | 0 | 1563 |
| TRINITY_DN2099_c0_g3_i1 | Contig_42147 | 96.618 | 828 | 14 | 6 | 33 | 858 | 1 | 816 | 0 | 1362 |
| TRINITY_DN21245_c0_g1_i2 | Contig_40080 | 95.787 | 712 | 22 | 5 | 112 | 820 | 10 | 716 | 0 | 1142 |
|  | Contig_39917 | 93.902 | 738 | 15 | 18 | 1028 | 1745 | 562 | 1289 | 0 | 1086 |
|  | Contig_8627 | 97.647 | 595 | 11 | 3 | 147 | 739 | 676 | 83 | 0 | 1018 |
|  | Contig_6045 | 94.706 | 510 | 17 | 8 | 156 | 658 | 1 | 507 | 0 | 784 |
| TRINITY_DN22304_c0_g1_i1 | Contig_31725 | 98.011 | 2363 | 27 | 6 | 21 | 2364 | 1 | 2362 | 0 | 4085 |
| TRINITY_DN23830_c0_g1_i4 | Contig_12847 | 97.534 | 365 | 9 | 0 | 1 | 365 | 140 | 504 | 0 | 625 |
| TRINITY_DN23865_c0_g1_i1 | Contig_9007 | 98.928 | 1492 | 13 | 2 | 1 | 1489 | 1683 | 192 | 0 | 2663 |
| TRINITY_DN248314_c0_g1_i1 | Contig_19672 | 93.928 | 774 | 14 | 14 | 510 | 1278 | 12 | 757 | 0 | 1138 |
| TRINITY_DN25090_c0_g1_i4 | Contig_13779 | 91.339 | 508 | 44 | 0 | 1 | 508 | 140 | 647 | 0 | 695 |
|  | Contig_11327 | 81.977 | 516 | 83 | 7 | 1 | 508 | 292 | 805 | 4.24E-122 | 429 |
|  | Contig_13041 | 85.342 | 307 | 41 | 4 | 204 | 508 | 11 | 315 | 1.24E-87 | 315 |
|  | Contig_9434 | 77.825 | 469 | 95 | 8 | 46 | 508 | 1876 | 1411 | 1.26E-77 | 281 |
|  | Contig_40910 | 74.536 | 377 | 63 | 22 | 1 | 349 | 683 | 1054 | 3.73E-33 | 134 |
| TRINITY_DN25744_c0_g1_i5 | Contig_28281 | 87.618 | 953 | 110 | 6 | 4 | 952 | 2036 | 1088 | 0 | 1099 |
|  | Contig_28281 | 92.34 | 705 | 50 | 3 | 4 | 706 | 1784 | 1082 | 0 | 1000 |
| TRINITY_DN25758_c1_g2_i1 | Contig_46261 | 95.982 | 224 | 2 | 2 | 1 | 223 | 218 | 1 | 1.12E-100 | 357 |
| TRINITY_DN273_c8_g1_i1 | Contig_1973 | 92.995 | 828 | 14 | 4 | 103 | 929 | 880 | 96 | 0 | 1168 |
| TRINITY_DN2969_c2_g1_i10 | Contig_36148 | 98.963 | 482 | 5 | 0 | 1 | 482 | 590 | 1071 | 0 | 863 |
| TRINITY_DN29716_c0_g1_i1 | Contig_30333 | 97.053 | 1459 | 20 | 10 | 242 | 1692 | 1472 | 29 | 0 | 2435 |
| TRINITY_DN30556_c0_g1_i2 | Contig_36148 | 87.194 | 695 | 89 | 0 | 1 | 695 | 5119 | 4425 | 0 | 791 |
| TRINITY_DN3080_c1_g1_i9 | Contig_13041 | 91.321 | 265 | 23 | 0 | 1 | 265 | 52 | 316 | 5.15E-102 | 363 |
|  | Contig_13779 | 84.429 | 289 | 41 | 4 | 3 | 289 | 386 | 672 | 1.48E-77 | 281 |
| TRINITY_DN30869_c0_g2_i1 | Contig_43923 | 98.178 | 3183 | 54 | 2 | 89 | 3271 | 3308 | 130 | 0 | 5553 |
| TRINITY_DN31999_c1_g1_i1 | Contig_44356 | 99.294 | 1275 | 9 | 0 | 55 | 1329 | 1 | 1275 | 0 | 2305 |
| TRINITY_DN33062_c0_g1_i1 | Contig_7912 | 98.686 | 1598 | 21 | 0 | 592 | 2189 | 1605 | 8 | 0 | 2835 |
| TRINITY_DN3478_c0_g1_i1 | Contig_35869 | 98.319 | 1785 | 20 | 2 | 15 | 1793 | 1804 | 24 | 0 | 3121 |
| TRINITY_DN3623_c1_g1_i2 | Contig_37617 | 93.437 | 1219 | 62 | 10 | 397 | 1602 | 436 | 1649 | 0 | 1792 |
| TRINITY_DN37579_c1_g1_i1 | Contig_32496 | 98.626 | 1310 | 15 | 2 | 91 | 1398 | 1342 | 34 | 0 | 2316 |
| TRINITY_DN37965_c1_g1_i5 | Contig_35910 | 97.18 | 1525 | 31 | 3 | 10 | 1534 | 1 | 1513 | 0 | 2567 |
| TRINITY_DN392408_c0_g1_i1 | Contig_30329 | 99.796 | 489 | 1 | 0 | 1 | 489 | 2029 | 2517 | 0 | 898 |
| TRINITY_DN42156_c0_g1_i7 | Contig_36148 | 96.552 | 464 | 16 | 0 | 1 | 464 | 1980 | 2443 | 0 | 769 |
|  | Contig_10463 | 85.991 | 464 | 59 | 4 | 1 | 464 | 507 | 964 | 4.86E-141 | 492 |
| TRINITY_DN4236_c0_g1_i1 | Contig_3066 | 96.939 | 1470 | 32 | 8 | 1 | 1468 | 1973 | 3431 | 0 | 2453 |
| TRINITY_DN4241_c0_g1_i1 | Contig_43825 | 95.519 | 491 | 18 | 2 | 40 | 526 | 524 | 34 | 0 | 782 |
| TRINITY_DN4520_c0_g1_i2 | Contig_44275 | 95.751 | 1812 | 49 | 9 | 4 | 1788 | 2971 | 1161 | 0 | 2894 |
| TRINITY_DN47850_c0_g1_i1 | Contig_6914 | 98.483 | 923 | 11 | 2 | 273 | 1192 | 1 | 923 | 0 | 1624 |
| TRINITY_DN51440_c0_g1_i1 | Contig_31651 | 95.773 | 1112 | 46 | 1 | 278 | 1389 | 1 | 1111 | 0 | 1792 |
| TRINITY_DN5447_c0_g1_i3 | Contig_16225 | 97.766 | 1656 | 37 | 0 | 17 | 1672 | 1671 | 16 | 0 | 2854 |
| TRINITY_DN5631_c0_g1_i2 | Contig_23206 | 95.56 | 518 | 21 | 2 | 3 | 519 | 815 | 1331 | 0 | 828 |
|  | Contig_46256 | 91.195 | 477 | 36 | 4 | 14 | 486 | 608 | 1082 | 0 | 643 |
|  | Contig_41494 | 91.453 | 468 | 36 | 4 | 3 | 469 | 927 | 1391 | 0 | 640 |
|  | Contig_47550 | 88.497 | 539 | 46 | 7 | 2 | 540 | 523 | 1 | 0 | 638 |
|  | Contig_44174 | 91.102 | 472 | 36 | 5 | 2 | 469 | 1024 | 1493 | 0 | 634 |
| TRINITY_DN5786_c0_g1_i1 | Contig_30994 | 98.407 | 1318 | 11 | 3 | 80 | 1391 | 1440 | 127 | 0 | 2309 |
| TRINITY_DN61746_c0_g1_i5 | Contig_36148 | 93.867 | 375 | 23 | 0 | 1 | 375 | 262 | 636 | 2.26E-163 | 566 |
| TRINITY_DN6454_c0_g1_i1 | Contig_41918 | 97.11 | 692 | 6 | 7 | 90 | 775 | 728 | 45 | 0 | 1155 |
| TRINITY_DN674_c0_g1_i8 | Contig_23206 | 95.506 | 534 | 18 | 3 | 25 | 557 | 3 | 531 | 0 | 848 |
|  | Contig_41494 | 92.541 | 362 | 20 | 3 | 60 | 418 | 1 | 358 | 4.51E-147 | 512 |
|  | Contig_44174 | 86.286 | 350 | 33 | 10 | 116 | 455 | 179 | 523 | 7.92E-110 | 388 |
|  | Contig_46256 | 97.436 | 78 | 2 | 0 | 480 | 557 | 1 | 78 | 4.10E-33 | 134 |
|  | Contig_47550 | 98.462 | 65 | 1 | 0 | 493 | 557 | 870 | 806 | 1.48E-27 | 115 |
| TRINITY_DN6819_c0_g1_i1 | Contig_36030 | 97.778 | 1305 | 24 | 3 | 3 | 1303 | 1304 | 1 | 0 | 2244 |
| TRINITY_DN6840_c0_g1_i1 | Contig_35944 | 87.5 | 200 | 25 | 0 | 3847 | 4046 | 97 | 296 | 1.07E-61 | 231 |
| TRINITY_DN6969_c1_g1_i2 | Contig_10501 | 96.568 | 1282 | 26 | 5 | 173 | 1451 | 11 | 1277 | 0 | 2108 |
| TRINITY_DN6986_c0_g1_i1 | Contig_42362 | 98.306 | 1889 | 16 | 2 | 23 | 1905 | 82 | 1960 | 0 | 3297 |
| TRINITY_DN70086_c0_g1_i6 | Contig_36148 | 87.78 | 491 | 56 | 4 | 1 | 489 | 4132 | 4620 | 6.40E-165 | 571 |
| TRINITY_DN726_c0_g1_i1 | Contig_7184 | 96.211 | 1610 | 30 | 6 | 983 | 2566 | 1668 | 64 | 0 | 2606 |
|  | Contig_17293 | 82.667 | 75 | 12 | 1 | 1988 | 2061 | 643 | 569 | 7.56E-12 | 65.8 |
| TRINITY_DN7272_c1_g1_i1 | Contig_5342 | 95.782 | 569 | 20 | 3 | 1 | 567 | 567 | 1 | 0 | 915 |
| TRINITY_DN73988_c0_g1_i5 | Contig_36148 | 95.977 | 348 | 14 | 0 | 1 | 348 | 753 | 1100 | 2.09E-163 | 566 |
| TRINITY_DN7661_c0_g2_i3 | Contig_45427 | 97.566 | 493 | 12 | 0 | 240 | 732 | 1658 | 1166 | 0 | 845 |
| TRINITY_DN7776_c0_g1_i1 | Contig_11740 | 95.385 | 195 | 5 | 3 | 1 | 191 | 496 | 690 | 1.13E-85 | 307 |
| TRINITY_DN7778_c0_g1_i1 | Contig_42743 | 97.856 | 2052 | 30 | 3 | 2 | 2045 | 1 | 2046 | 0 | 3533 |
| TRINITY_DN7863_c0_g1_i7 | Contig_28513 | 94.322 | 1180 | 25 | 9 | 24 | 1173 | 1360 | 193 | 0 | 1770 |
| TRINITY_DN8282_c0_g2_i1 | Contig_43155 | 97.826 | 1058 | 16 | 5 | 95 | 1146 | 1058 | 2 | 0 | 1820 |
| TRINITY_DN8472_c0_g1_i3 | Contig_8252 | 96.891 | 2798 | 64 | 10 | 6 | 2789 | 6070 | 3282 | 0 | 4663 |
| TRINITY_DN8537_c0_g1_i2 | Contig_7128 | 98.424 | 1904 | 27 | 2 | 51 | 1954 | 28 | 1928 | 0 | 3347 |
| TRINITY_DN9140_c0_g1_i1 | Contig_18078 | 98.963 | 2025 | 20 | 1 | 3 | 2026 | 1 | 2025 | 0 | 3622 |
| TRINITY_DN9766_c0_g1_i1 | Contig_32689 | 97.804 | 1184 | 26 | 0 | 112 | 1295 | 1370 | 187 | 0 | 2043 |
| TRINITY_DN992_c0_g1_i3 | Contig_17293 | 96.148 | 2025 | 52 | 6 | 61 | 2084 | 2022 | 23 | 0 | 3284 |
|  | Contig_7184 | 81.333 | 75 | 13 | 1 | 1464 | 1538 | 642 | 569 | 2.70E-10 | 60.2 |
| TRINITY_DN9942_c0_g1_i3 | Contig_16588 | 97.18 | 1064 | 29 | 1 | 103 | 1165 | 3950 | 2887 | 0 | 1797 |
| TRINITY_DN9953_c0_g1_i1 | Contig_17323 | 98.175 | 1151 | 21 | 0 | 5 | 1155 | 10 | 1160 | 0 | 2010 |
