# Supplementary material for: Gene expression profiles of Japanese precious coral Corallium japonicum during gametogenesis
Source: PeerJ. 2024 Apr 16;12:e17182. doi: 10.7717/peerj.17182 (PMC11027906; doi:10.7717/peerj.17182)
Supplement: Supplemental Information 9 [file peerj-12-17182-s009.docx]

**Supplemental Table 4A**. Results of the similarity search for analogous protein sequences (blastp) for female de novo assembled transcripts and protein annotation (i.e., Molecular function and Biological process) from Uniprot database. Data only shows the most significant hits (<0.001).

| **No.** | **Category** | **qaccver** | **saccver** | **pident** | **length** | **evalue** | **bitscore** | **Protein** | **Molecular Function** | **Biological Process** |
| --- | --- | --- | --- | --- | --- | --- | --- | --- | --- | --- |
| 1 | Cell cycle and division | TRINITY_DN15375_c0_g2_i1 | sp\|Q3TZI6\|CCNJ_MOUSE | 46.1 | 254 | 3.59E-68 | 227 | Cyclin-J | Cyclin-dependent protein serine/threonine kinase regulator activity | mitotic cell cycle phase transition |
| 2 |  | TRINITY_DN6454_c0_g1_i1 | sp\|Q6I6F4\|BIRC5_FELCA | 45.2 | 126 | 7.14E-32 | 117 | Baculoviral IAP repeat-containing protein 5 | cysteine-type endopeptidase inhibitor activity cysteine-type endopeptidase inhibitor activity involved in apoptotic process microtubule binding protein homodimerization activity tubulin binding zinc ion binding | apoptotic process cell division chromosome segregation establishment of chromosome localization G2/M transition of mitotic cell cycle meiosis I microtubule cytoskeleton organization mitotic cytokinesis mitotic spindle assembly checkpoint signaling negative regulation of apoptotic process negative regulation of cysteine-type endopeptidase activity involved in apoptotic process negative regulation of DNA-templated transcription negative regulation of neuron apoptotic process positive regulation of exit from mitosis positive regulation of mitotic cell cycle protein phosphorylation |
| 3 |  | TRINITY_DN1115_c0_g1_i4 | sp\|Q91879\|CKS2_XENLA | 70.3 | 74 | 1.05E-30 | 108 | Cyclin-dependent kinases regulatory subunit 2 | cyclin-dependent protein serine/threonine kinase regulator activity | Cell cycle Cell division |
| 4 |  | TRINITY_DN3478_c0_g1_i1 | sp\|P39963\|CCNB3_CHICK | 45 | 407 | 3.41E-95 | 300 | G2/mitotic-specific cyclin-B3 | cyclin-dependent protein serine/threonine kinase regulator activity | cell division mitotic cell cycle phase transition |
| 5 |  | TRINITY_DN103939_c0_g1_i7 | sp\|P51954\|NEK1_MOUSE | 50.2 | 267 | 3.69E-80 | 281 | Serine/threonine-protein kinase Nek1 | 14-3-3 protein binding ATP binding kinase activity metal ion binding protein kinase activity protein serine kinase activity protein serine/threonine kinase activity protein tyrosine kinase activity | cell cycle cell division cilium assembly DNA damage response kidney development multicellular organism growth phosphorylation regulation of DNA damage checkpoint response to ionizing radiation spermatogenesis |
| 6 |  | TRINITY_DN4929_c1_g1_i2 | sp\|Q99741\|CDC6_HUMAN | 51.7 | 408 | 2.18E-136 | 419 | Cell division control protein 6 homolog | ATP binding ATP hydrolysis activity kinase binding nucleotide binding | cell division cellular response to angiotensin cellular response to vasopressin DNA replication checkpoint signaling DNA replication initiation mitotic DNA replication checkpoint signaling negative regulation of cell population proliferation negative regulation of DNA replication positive regulation of chromosome segregation positive regulation of cytokinesis positive regulation of fibroblast proliferation regulation of cyclin-dependent protein serine/threonine kinase activity regulation of mitotic metaphase/anaphase transition traversing start control point of mitotic cell cycle |
| 7 |  | TRINITY_DN6986_c0_g1_i1 | sp\|P24861\|CCNA_PATVU | 49.2 | 419 | 4.65E-106 | 330 | G2/mitotic-specific cyclin-A | cyclin-dependent protein serine/threonine kinase regulator activity | cell division mitotic cell cycle phase transition |
| 8 |  | TRINITY_DN726_c0_g1_i1 | sp\|P15206\|CCNB_MARGL | 46.8 | 391 | 2.13E-94 | 305 | G2/mitotic-specific cyclin-B | cyclin-dependent protein serine/threonine kinase regulator activity | cell division mitotic cell cycle phase transition |
| 9 |  | TRINITY_DN992_c0_g1_i3 | sp\|P24862\|CCNB_PATVU | 41 | 402 | 2.34E-86 | 280 | G2/mitotic-specific cyclin-B | cyclin-dependent protein serine/threonine kinase regulator activity | cell division mitotic cell cycle phase transition |
| 10 |  | TRINITY_DN4520_c0_g1_i2 | sp\|P15206\|CCNB_MARGL | 44.3 | 393 | 3.34E-82 | 266 | G2/mitotic-specific cyclin-B | cyclin-dependent protein serine/threonine kinase regulator activity | cell division mitotic cell cycle phase transition |
| 11 |  | TRINITY_DN5786_c0_g1_i1 | sp\|P23437\|CDK2_XENLA | 73.6 | 295 | 1.99E-160 | 458 | Cyclin-dependent kinase 2 | ATP binding cyclin-dependent protein kinase activity cyclin-dependent protein serine/threonine kinase activity protein serine kinase activity | cell cycle cell division protein phosphorylation |
| 12 |  | TRINITY_DN47850_c0_g1_i1 | sp\|P56616\|UBE2C_XENLA | 65.1 | 175 | 1.45E-71 | 226 | Ubiquitin-conjugating enzyme E2 C | ATP binding ubiquitin conjugating enzyme activity | anaphase-promoting complex-dependent catabolic process cell division exit from mitosis free ubiquitin chain polymerization protein K-linked ubiquitination ubiquitin-dependent protein catabolic process |
| 13 |  | TRINITY_DN30869_c0_g2_i1 | sp\|E7F6T8\|UBP37_DANRE | 27.3 | 722 | 1.07E-48 | 191 | Ubiquitin carboxyl-terminal hydrolase 37 | cysteine-type deubiquitinase activity cysteine-type endopeptidase activity | cell division central nervous system morphogenesis cranial skeletal system development G/S transition of mitotic cell cycle protein K-linked deubiquitination protein K48-linked deubiquitination |
| 14 |  | TRINITY_DN6947_c0_g1_i1 | sp\|Q7ZUY3\|H2AX_DANRE | 73.9 | 142 | 1.57E-62 | 198 | Histone H2AX | DNA binding protein heterodimerization activity structural constituent of chromatin | DNA damage response DNA recombination DNA repair meiotic cell cycle |
| 15 |  | TRINITY_DN33062_c0_g1_i1 | sp\|Q9I9A7\|CDT1_XENLA | 40.2 | 391 | 1.22E-68 | 240 | DNA replication factor Cdt1 | chromatin DNA binding | DNA replication preinitiation complex assembly regulation of DNA-templated DNA replication initiationterPro |
| 16 |  | TRINITY_DN9140_c0_g1_i1 | sp\|A6H8I0\|UBP22_DANRE | 57.5 | 534 | 2.38E-208 | 596 | Ubiquitin carboxyl-terminal hydrolase 22 | cysteine-type deubiquitinase activity zinc ion binding | cell cycle chromatin organization proteolysis |
| 17 |  | TRINITY_DN48905_c0_g1_i10 | sp\|D3Z3K2\|CIP1_MOUSE | 45.8 | 179 | 4.29E-44 | 159 | E3 ubiquitin-protein ligase CCNB1IP1 | identical protein binding metal ion binding ubiquitin protein ligase activity ubiquitin-protein transferase activity | blastocyst formation chiasma assembly protein ubiquitination reciprocal meiotic recombination spermatid developmen |
| 18 |  | TRINITY_DN5533_c0_g1_i5 | sp\|Q9GRC0\|MOS_PATPE | 45.3 | 267 | 1.81E-69 | 231 | Serine/threonine-protein kinase mos | ATP binding MAP kinase kinase kinase activity protein serine kinase activity | female gamete generation female meiosis II negative regulation of mitotic cell cycle phosphorylation |
| 19 |  | TRINITY_DN6145_c1_g2_i3 | sp\|Q8N9W6\|BOLL_HUMAN | 43.7 | 87 | 1.50E-12 | 71.2 | Protein boule-like | identical protein binding mRNA 3'-UTR binding translation activator activity | 3'-UTR-mediated mRNA stabilization cell differentiation meiotic cell cycle positive regulation of translational initiation spermatogenesis |
| 20 |  | TRINITY_DN11811_c0_g1_i1 | sp\|P30740\|ILEU_HUMAN | 42.6 | 296 | 3.20E-73 | 238 | Leukocyte elastase inhibitor | peptidase inhibitor activity serine-type endopeptidase inhibitor activity Serine protease inhibitor | negative regulation of endopeptidase activity negative regulation of interleukin- beta production type B pancreatic cell proliferation |
| 21 | Metabolism and transport | TRINITY_DN10917_c0_g1_i2 | sp\|Q21565\|AMT3_CAEEL | 50.8 | 303 | 6.59E-87 | 291 | Putative ammonium transporter 3 | ammonium transmembrane transporter activity | Ammonium homeostasis Ammonium transmembrane transport |
| 21 |  | TRINITY_DN7661_c0_g2_i3 | sp\|P54147\|Y108_SYNY3 | 39.8 | 88 | 2.46E-09 | 60.8 | Putative ammonium transporter sll0108 | ammonium transmembrane transporter activity | Ammonium homeostasis Ammonium transmembrane transport |
| 23 |  | TRINITY_DN2969_c2_g1_i10 | sp\|P12259\|FA5_HUMAN | 39.3 | 150 | 1.28E-20 | 90.9 | Coagulation factor V | copper ion binding | blood circulation blood coagulation response to vitamin K |
| 24 |  | TRINITY_DN61746_c0_g1_i5 | sp\|Q9TU53\|CUBN_CANLF | 53.1 | 32 | 7.35E-05 | 44.3 | Cubilin | calcium ion binding cobalamin binding | Cholesterol metabolic process protein transport |
| 25 |  | TRINITY_DN12587_c0_g1_i1 | sp\|Q1W694\|B1Q_LOXIN | 37 | 289 | 1.45E-57 | 191 | Dermonecrotic toxin LiSicTox-betaID1 | phospholipase activity phosphoric diester hydrolase activity toxin activity | envenomation resulting in induction of edema in another organism killing of cells of another organism lipid catabolic process phospholipid metabolic process |
| 26 |  | TRINITY_DN7586_c0_g1_i1 | sp\|Q98SN9\|S61A1_ONCMY | 80.4 | 51 | 3.06E-23 | 94.7 | Protein transport protein Sec61 subunit alpha isoform A | Developmental protein | pronephric nephron development protein targeting to ER |
| 27 |  | TRINITY_DN392408_c0_g1_i1 | sp\|P97329\|KI20A_MOUSE | 49.6 | 127 | 6.05E-37 | 137 | Kinesin-like protein KIF20A | ATP binding ATP hydrolysis activity microtubule binding microtubule motor activity protein kinase binding | microtubule bundle formation microtubule-based movement midbody abscission mitotic cytokinesis protein transport regulation of cytokinesis |
| 28 |  | TRINITY_DN4578_c0_g1_i1 | sp\|O35343\|IMA3_MOUSE | 59.2 | 530 | 4.92E-99 | 318 | Importin subunit alpha-3 | nuclear import signal receptor activity nuclear localization sequence binding | dopamine secretion gene expression NLS-bearing protein import into nucleus protein import into nucleus response to hydrogen peroxide |
| 29 |  | TRINITY_DN29796_c0_g1_i1 | sp\|P97852\|DHB4_RAT | 41.3 | 126 | 1.69E-24 | 101 | Peroxisomal multifunctional enzyme type | enoyl-CoA hydratase activity estradiol 17-beta-dehydrogenase [NAD(P)] activity identical protein binding isomerase activity protein homodimerization activity testosterone dehydrogenase [NAD(P)] activity | androgen metabolic process cellular response to organic cyclic compound cholesterol metabolic process estrogen metabolic process fatty acid beta-oxidation medium-chain fatty-acyl-CoA metabolic process response to organic cyclic compound response to steroid hormone response to xenobiotic stimulus Sertoli cell development very long-chain fatty acid metabolic process very long-chain fatty-acyl-CoA metabolic process |
| 30 |  | TRINITY_DN13209_c0_g1_i5 | sp\|Q7Z020\|TRPA1_DROME | 24.3 | 321 | 1.57E-05 | 53.1 | Transient receptor potential cation channel subfamily A member 1 | calcium channel activity ligand-gated monoatomic ion channel activity monoatomic cation channel activity temperature-gated cation channel activity | calcium ion transport cellular response to heat detection of chemical stimulus involved in sensory perception of bitter taste detection of chemical stimulus involved in sensory perception of pain detection of temperature stimulus involved in sensory perception of pain detection of temperature stimulus involved in thermoception mechanosensory behavior monoatomic cation transport negative phototaxis neuronal signal transduction phototransduction positive regulation of calcium-mediated signaling response to heat response to light stimulus temperature compensation of the circadian clock thermosensory behavior thermotaxis |
| 31 |  | TRINITY_DN7778_c0_g1_i1 | sp\|I6V1W0\|BMBL_DANRE | 36.5 | 384 | 3.02E-72 | 248 | Protein brambleberry |  | karyomere membrane fusion pronuclear fusion |
| 32 |  | TRINITY_DN48658_c0_g1_i1 | sp\|Q8C5H1\|ANO4_MOUSE | 37 | 864 | 1.25E-154 | 484 | Anoctamin-4 | chloride channel activity phospholipid scramblase activity protein dimerization activity | calcium activated galactosylceramide scrambling calcium activated phosphatidylcholine scrambling calcium activated phosphatidylserine scrambling chloride transmembrane transport chloride transport establishment of localization in cell |
| 33 |  | TRINITY_DN12942_c1_g1_i7 | sp\|Q8CFM6\|STAB2_RAT | 54.5 | 44 | 2.51E-04 | 47.4 | Stabilin-2 | calcium ion binding cargo receptor activity hyaluronic acid binding low-density lipoprotein particle binding low-density lipoprotein particle receptor activity | cell adhesion defense response to bacterium defense response to Gram-positive bacterium endocytosis hyaluronan catabolic process receptor-mediated endocytosis |
| 34 |  | TRINITY_DN6840_c0_g1_i1 | sp\|Q98930\|SORL_CHICK | 55.3 | 38 | 2.07E-05 | 53.1 | Sortilin-related receptor | Receptor | endocytosis post-Golgi vesicle-mediated transport protein retention in Golgi apparatus protein targeting protein targeting to lysosome |
| 35 |  | TRINITY_DN3270_c0_g1_i1 | sp\|O93477\|SAHHB_XENLA | 78.4 | 125 | 4.34E-64 | 203 | Adenosylhomocysteinase B | adenosylhomocysteinase activity NAD binding | One-carbon metabolism |
| 36 |  | TRINITY_DN5075_c0_g1_i6 | sp\|P15870\|H1D_STRPU | 41.3 | 104 | 1.90E-09 | 60.5 | Histone H1-delta | double-stranded DNA binding nucleosomal DNA binding structural constituent of chromatin | chromosome condensation negative regulation of DNA recombination nucleosome assembly |
| 37 | Genetic Processes | TRINITY_DN21245_c0_g1_i2 | sp\|P35061\|H2A_ACRFO | 89.5 | 124 | 3.70E-67 | 217 | Histone H2A | DNA binding protein heterodimerization activity structural constituent of chromatin |  |
| 38 |  | TRINITY_DN25744_c0_g1_i5 | sp\|P31509\|ESCA_CRYAO | 55.6 | 27 | 1.72E-04 | 42 | Escargot/snail protein homolog | DNA binding metal ion binding |  |
| 39 |  | TRINITY_DN23865_c0_g1_i1 | sp\|Q9I8B3\|RB24B_XENLA | 46.9 | 143 | 2.13E-31 | 124 | RNA-binding protein 24-B | mRNA 3'-UTR AU-rich region binding mRNA 3'-UTR binding mRNA CDS binding pre-mRNA intronic binding sequence-specific mRNA binding | 3'-UTR-mediated mRNA destabilization cell differentiation DNA damage response mRNA destabilization mRNA processing mRNA stabilization negative regulation of cytoplasmic translation positive regulation of 3'-UTR-mediated mRNA stabilization positive regulation of myoblast differentiation positive regulation of myotube differentiation positive regulation of skeletal muscle fiber differentiation positive regulation of stem cell differentiation regulation of alternative mRNA splicing, via spliceosome regulation of mRNA stability regulation of myotube differentiation RNA splicing |
| 40 |  | TRINITY_DN8472_c0_g1_i3 | sp\|Q803U7\|EXO1_DANRE | 50.1 | 347 | 5.50E-91 | 311 | Exonuclease 1 | 5'-3' DNA exonuclease activity 5'-flap endonuclease activity DNA binding flap endonuclease activity metal ion binding single-stranded DNA 5'-3' DNA exonuclease activity | DNA recombination mismatch repair |
| 41 |  | TRINITY_DN10744_c0_g1_i3 | sp\|Q1ZZU3\|SWI5_HUMAN | 54.1 | 74 | 2.49E-14 | 73.9 | DNA repair protein SWI5 homolog |  | cellular response to ionizing radiation double-strand break repair via homologous recombination |
| 42 |  | TRINITY_DN4236_c0_g1_i1 | sp\|P04047\|KITH_CHICK | 62.8 | 191 | 3.08E-80 | 252 | Thymidine kinase, cytosolic | ATP binding identical protein binding thymidine kinase activity zinc ion binding | DNA biosynthetic process phosphorylation protein homotetramerization thymidine metabolic process |
| 43 |  | TRINITY_DN10913_c0_g1_i15 | sp\|O00142\|KITM_HUMAN | 49.3 | 209 | 2.29E-66 | 216 | Thymidine kinase 2, mitochondrial | ATP binding deoxycytidine kinase activity deoxynucleoside kinase activity nucleoside kinase activity thymidine kinase activity | deoxycytidine metabolic process DNA biosynthetic process nucleobase-containing compound metabolic process phosphorylation pyrimidine nucleoside salvage thymidine metabolic process |
| 44 |  | TRINITY_DN6819_c0_g1_i1 | sp\|Q14493\|SLBP_HUMAN | 38.2 | 207 | 9.16E-25 | 106 | Histone RNA hairpin-binding protein | histone pre-mRNA DCP binding histone pre-mRNA stem-loop binding identical protein binding mRNA binding RNA binding | cap-dependent translational initiation mRNA 3'-end processing by stem-loop binding and cleavage mRNA transport |
| 45 |  | TRINITY_DN3881_c0_g1_i1 | sp\|Q9WV03\|FA50A_MOUSE | 70.7 | 41 | 7.54E-09 | 54.3 | Protein FAM50A |  | chromatin organization mRNA processingS regulation of RNA splicing RNA splicing |
| 46 |  | TRINITY_DN42270_c0_g1_i1 | sp\|Q0VDT2\|ZN367_MOUSE | 50.7 | 229 | 1.37E-30 | 126 | Zinc finger protein 367 | DNA-binding transcription factor activity DNA-binding transcription factor activity, RNA polymerase II-specific metal ion binding RNA polymerase II cis-regulatory region sequence-specific DNA binding | regulation of transcription by RNA polymerase II |
| 47 |  | TRINITY_DN12746_c0_g1_i1 | sp\|Q18248\|DMD4_CAEEL | 34.3 | 178 | 7.39E-20 | 92.4 | Doublesex- and mab-3-related transcription factor dmd-4 | Function DNA-binding transcription factor activity, RNA polymerase II-specific metal ion binding RNA polymerase II cis-regulatory region sequence-specific DNA binding ubiquitin binding | regulation of transcription by RNA polymerase II sex differentiation synapse pruning |
| 48 |  | TRINITY_DN14879_c0_g1_i8 | sp\|Q9GKU5\|HDA11_MACFA | 64.1 | 323 | 7.25E-153 | 441 | Histone deacetylase 11 | DNA-binding transcription factor binding histone deacetylase activity | Chromatin organization |
| 49 |  | TRINITY_DN16128_c1_g1_i6 | sp\|Q9QYE3\|BC11A_MOUSE | 60.7 | 28 | 1.64E-05 | 46.2 | B-cell lymphoma/leukemia 11A | DNA-binding transcription factor activity DNA-binding transcription factor binding DNA-binding transcription repressor activity, RNA polymerase II-specific metal ion binding protein heterodimerization activity protein homodimerization activity protein kinase binding RNA polymerase II cis-regulatory region sequence-specific DNA binding transcription coregulator activity transcription corepressor activity transcription regulatory region nucleic acid binding | B cell differentiation gene expression negative regulation of branching morphogenesis of a nerve negative regulation of collateral sprouting negative regulation of dendrite development negative regulation of dendrite extension negative regulation of gene expression negative regulation of neuron projection development negative regulation of neuron remodeling negative regulation of transcription by RNA polymerase II positive regulation of collateral sprouting positive regulation of gene expression positive regulation of neuron projection development protein sumoylation regulation of dendrite development regulation of transcription by RNA polymerase II T cell differentiation |
| 50 |  | TRINITY_DN3080_c1_g1_i9 | sp\|Q9QYE3\|BC11A_MOUSE | 58.6 | 29 | 1.03E-04 | 45.8 | B-cell lymphoma/leukemia 11A | DNA-binding transcription factor activity DNA-binding transcription factor binding DNA-binding transcription repressor activity, RNA polymerase II-specific metal ion binding protein heterodimerization activity protein homodimerization activity protein kinase binding RNA polymerase II cis-regulatory region sequence-specific DNA binding transcription coregulator activity transcription corepressor activity transcription regulatory region nucleic acid binding | B cell differentiation gene expression negative regulation of branching morphogenesis of a nerve negative regulation of collateral sprouting negative regulation of dendrite development negative regulation of dendrite extension negative regulation of gene expression negative regulation of neuron projection development negative regulation of neuron remodeling negative regulation of transcription by RNA polymerase II positive regulation of collateral sprouting positive regulation of gene expression positive regulation of neuron projection development protein sumoylation regulation of dendrite development regulation of transcription by RNA polymerase II T cell differentiation |
| 51 |  | TRINITY_DN353237_c0_g1_i1 | sp\|O01727\|RS6_BRAFL | 91.2 | 68 | 6.96E-38 | 128 | Small ribosomal subunit protein eS6 | structural constituent of ribosome | translation |
| 52 |  | TRINITY_DN26128_c1_g1_i1 | sp\|Q3ZBH8\|RS20_BOVIN | 92.5 | 67 | 3.31E-38 | 125 | Small ribosomal subunit protein uS10 | MDM2/MDM4 family protein binding RNA binding structural constituent of ribosome ubiquitin ligase inhibitor activity | positive regulation of signal transduction by p53 class mediator translation |
| 53 |  | TRINITY_DN17267_c5_g1_i1 | sp\|Q90YU9\|RL18A_ICTPU | 71.4 | 28 | 2.58E-07 | 48.5 | Large ribosomal subunit protein eL20 | structural constituent of ribosome | translation |
| 54 | Cell Signaling and Response | TRINITY_DN1441_c0_g1_i1 | sp\|P08419\|CEL2A_PIG | 45.3 | 247 | 4.05E-54 | 193 | Chymotrypsin-like elastase family member 2A | endopeptidase activity serine-type endopeptidase activity | Insulin catabolic process proteolysis regulation of insulin secretion regulation of platelet aggregation response to insulin |
| 55 |  | TRINITY_DN16152_c0_g1_i2 | sp\|Q28295\|VWF_CANLF | 30.4 | 368 | 7.45E-40 | 157 | von Willebrand factor | collagen binding identical protein binding immunoglobulin binding integrin binding protease binding protein-folding chaperone binding | blood coagulation cell adhesion cell-substrate adhesion hemostasis platelet activation |
| 56 |  | TRINITY_DN42156_c0_g1_i7 | sp\|P80012\|VWF_BOVIN | 32.1 | 140 | 4.26E-15 | 74.7 | von Willebrand factor | collagen binding identical protein binding immunoglobulin binding integrin binding protease binding protein-folding chaperone binding | blood coagulation cell adhesion cell-substrate adhesion hemostasis platelet activation |
| 57 |  | TRINITY_DN73988_c0_g1_i5 | sp\|O43854\|EDIL3_HUMAN | 34.9 | 106 | 4.23E-19 | 84.3 | EGF-like repeat and discoidin I-like domain-containing protein 3 | calcium ion binding integrin binding | cell adhesion positive regulation of cell-substrate adhesion |
| 58 |  | TRINITY_DN70086_c0_g1_i6 | sp\|O35474\|EDIL3_MOUSE | 38.9 | 149 | 1.22E-19 | 87.8 | EGF-like repeat and discoidin I-like domain-containing protein 3 | calcium ion binding | cell adhesion positive regulation of cell-substrate adhesion |
| 59 |  | TRINITY_DN30556_c0_g1_i2 | sp\|O43854\|EDIL3_HUMAN | 37.8 | 74 | 6.99E-11 | 64.7 | EGF-like repeat and discoidin I-like domain-containing protein 3 | calcium ion binding integrin binding | cell adhesion positive regulation of cell-substrate adhesion |
| 60 |  | TRINITY_DN29716_c0_g1_i1 | sp\|Q96HU8\|DIRA2_HUMAN | 40.3 | 191 | 6.65E-30 | 119 | GTP-binding protein | GTP-binding Nucleotide-binding | Signal transduction |
| 61 |  | TRINITY_DN16376_c0_g1_i6 | sp\|P78504\|JAG1_HUMAN | 51.3 | 39 | 2.04E-07 | 57 | Protein jagged-1 | calcium ion binding growth factor activity1  molecular adaptor activity Notch binding1  phospholipid binding structural molecule activity | angiogenesis  aorta morphogenesis aortic valve morphogenesis blood vessel remodeling cardiac neural crest cell development involved in outflow tract morphogenesis cardiac right ventricle morphogenesis cardiac septum morphogenesis cell fate determination  ciliary body morphogenesis distal tubule development endocardial cushion cell development endothelial cell differentiation  hemopoiesis  inhibition of neuroepithelial cell differentiation inner ear auditory receptor cell differentiation keratinocyte differentiation  loop of Henle development morphogenesis of an epithelial sheet myoblast differentiation  negative regulation of cell migration  negative regulation of cell-cell adhesion negative regulation of cell-matrix adhesion  negative regulation of endothelial cell differentiation negative regulation of fat cell differentiation negative regulation of neuron differentiation negative regulation of stem cell differentiation  nephron development nervous system development  neuroendocrine cell differentiation neuronal stem cell population maintenance Notch signaling involved in heart developmentSource:BHF-UCL Notch signaling pathway podocyte development positive regulation of cardiac epithelial to mesenchymal transitionSource:BHF-UCL positive regulation of myeloid cell differentiationSource:Ensembl positive regulation of Notch signaling pathway positive regulation of osteoblast differentiationSource:Ensembl positive regulation of transcription by RNA polymerase IISource:BHF-UCL pulmonary artery morphogenesisSource:BHF-UCL pulmonary valve morphogenesisSource:BHF-UCL2s regulation of cell population proliferation regulation of epithelial cell proliferationSource:Ensembl regulation of reproductive processSource:Ensembl response to muramyl dipeptideSource:Ensembl T cell mediated immunity |
| 62 |  | TRINITY_DN5741_c1_g1_i2 | sp\|Q29RU2\|OIT3_BOVIN | 43.1 | 123 | 1.89E-20 | 99.4 | Oncoprotein-induced transcript 3 protein | calcium ion binding |  |
| 63 |  | TRINITY_DN10396_c0_g1_i1 | sp\|Q8WUQ7\|CATIN_HUMAN | 94.1 | 17 | 4.61E-04 | 41.6 | Splicing factor Cactin | RNA binding | cellular response to interleukin- cellular response to lipopolysaccharide cellular response to tumor necrosis factor innate immune response mRNA cis splicing, via spliceosome mRNA splicing, via spliceosome negative regulation of canonical NF-kappaB signal transduction negative regulation of innate immune response negative regulation of interferon-beta production negative regulation of interleukin-8 production negative regulation of lipopolysaccharide-mediated signaling pathway  negative regulation of NF-kappaB transcription factor activity negative regulation of protein phosphorylation negative regulation of toll-like receptor signaling pathway negative regulation of tumor necrosis factor production negative regulation of type I interferon-mediated signaling pathway |
| 64 |  | TRINITY_DN8282_c0_g2_i1 | sp\|O15347\|HMGB3_HUMAN | 41 | 173 | 8.58E-32 | 122 | High mobility group protein B3 | DNA binding, bending double-stranded DNA binding four-way junction DNA binding RNA binding | DNA geometric change DNA recombination innate immune response regulation of transcription by RNA polymerase II |
| 65 |  | TRINITY_DN7791_c0_g1_i1 | sp\|P58911\|TX60A_PHYSE | 31.5 | 451 | 1.64E-70 | 239 | DELTA-alicitoxin-Pse2a | toxin activity | killing of cells of another organism |
| 66 |  | TRINITY_DN5458_c0_g1_i15 | sp\|Q5T700\|LRAD1_HUMAN | 30.3 | 145 | 8.71E-06 | 48.9 | Low-density lipoprotein receptor class A domain-containing protein 1 | receptor |  |
| 67 |  | TRINITY_DN16152_c0_g2_i2 | sp\|Q98UI9\|MUC5B_CHICK | 43 | 86 | 2.09E-14 | 75.5 | Mucin-5B | Virion binding | cholesterol homeostasis intestinal cholesterol absorption  macrophage activation involved in immune response |
| 68 | Cell Structure and Dynamics | TRINITY_DN3539_c1_g1_i1 | sp\|Q7Z6J4\|FGD2_HUMAN | 31.2 | 330 | 1.60E-33 | 140 | FYVE, RhoGEF and PH domain-containing protein 2 | guanyl-nucleotide exchange factor activity metal ion binding phosphatidylinositol phosphate binding small GTPase binding | actin cytoskeleton organization cytoskeleton organization filopodium assembly regulation of cell shape regulation of GTPase activity regulation of small GTPase mediated signal transduction |
| 69 |  | TRINITY_DN14509_c0_g1_i1 | sp\|Q0P4I1\|CEP44_XENTR | 45 | 109 | 4.08E-23 | 104 | Centrosomal protein of 44 kDa | microtubule binding | centriole replication, centriole-centriole cohesion and centrosome cycle |
| 70 |  | TRINITY_DN6124_c0_g1_i1 | sp\|Q557J6\|DBNL_DICDI | 59.5 | 37 | 4.17E-05 | 43.9 | Drebrin-like protein | actin binding actin filament binding myosin I heavy chain binding profilin binding protein kinase binding | actin filament bundle assembly aggregation involved in sorocarp development cell motility establishment or maintenance of cell polarity negative regulation of phagocytosis phagosome maturation pseudopodium assembly regulation of actin filament polymerization |
| 71 |  | TRINITY_DN11289_c0_g1_i1 | sp\|A4QNG1\|CC167_XENTR | 46.7 | 75 | 1.50E-12 | 64.3 | Coiled-coil domain-containing protein | membrane protein |  |
| 72 |  | TRINITY_DN32265_c0_g2_i1 | sp\|Q68CR1\|SE1L3_HUMAN | 29.5 | 946 | 5.48E-112 | 380 | Protein sel-1 homolog 3 | membrane protein |  |
| 73 | Enzymatic Reactions | TRINITY_DN52912_c0_g1_i1 | sp\|Q07973\|CP24A_HUMAN | 30.3 | 534 | 6.30E-58 | 204 | 1,25-dihydroxyvitamin D(3) 24-hydroxylase, mitochondrial | 1-alpha,25-dihydroxyvitamin D3 23-hydroxylase activity3s 1-alpha,25-dihydroxyvitamin D3 24-hydroxylase activity4s 25-hydroxycholecalciferol-23-hydroxylase activity3s 25-hydroxycholecalciferol-24-hydroxylase activity2s heme binding1  iron ion binding vitamin D 25-hydroxylase activity | osteoblast differentiation response to vitamin D vitamin D catabolic process5  vitamin D metabolic process vitamin D receptor signaling pathway vitamin metabolic process |
| 74 |  | TRINITY_DN22304_c0_g1_i1 | sp\|A6H611\|MIPEP_MOUSE | 47.7 | 654 | 2.48E-202 | 593 | Mitochondrial intermediate peptidase | Function endopeptidase activity metal ion binding metalloendopeptidase activity | peptide metabolic process protein processing involved in protein targeting to mitochondrion |
| 75 | Ubiquitin-Related Processes: | TRINITY_DN9045_c0_g1_i2 | sp\|Q9QXP6\|MKRN1_MOUSE | 63 | 27 | 2.68E-04 | 48.1 | E3 ubiquitin-protein ligase makorin-1 | chromatin binding metal ion binding ubiquitin protein ligase activity | cellular response to leukemia inhibitory factorSource:MGI protein ubiquitination |
| 76 |  | TRINITY_DN37965_c1_g1_i5 | sp\|Q96L50\|LLR1_HUMAN | 31.1 | 415 | 1.17E-39 | 152 | Leucine-rich repeat protein 1 |  | protein ubiquitination signal transduction |
| 77 |  | TRINITY_DN9305_c3_g1_i1 | sp\|G0S9J5\|RSP5_CHATD | 22.6 | 345 | 3.22E-04 | 48.9 | E3 ubiquitin-protein ligase RSP5 | ubiquitin protein ligase activity | protein ubiquitination ubiquitin-dependent protein catabolic process |
| 78 |  | TRINITY_DN99502_c0_g1_i3 | sp\|Q7ZW16\|RNF41_DANRE | 33.1 | 127 | 5.44E-09 | 59.3 | E3 ubiquitin-protein ligase NRDP1 | ubiquitin protein ligase activity ubiquitin-protein transferase activity zinc ion binding | extrinsic apoptotic signaling pathway melanocyte differentiation protein polyubiquitination protein ubiquitination |
| 79 | Light Production | TRINITY_DN3623_c1_g1_i2 | sp\|P05938\|LBP_RENRE | 41.3 | 184 | 4.30E-38 | 141 | Luciferin-binding protein | Photoprotein | Luminescence |
| 80 | Growth and development | TRINITY_DN37579_c1_g1_i1 | sp\|P48970\|UNIV_STRPU | 31.7 | 287 | 4.53E-32 | 130 | Univin | Cytokine Growth factor activity |  |
| 81 |  | TRINITY_DN36054_c0_g1_i2 | sp\|Q9QXP4\|DONS_MOUSE | 33.8 | 284 | 2.76E-47 | 173 | Protein downstream neighbor of Son | Developmental protein |  |
| 82 | Unknown biological process | TRINITY_DN12116_c3_g2_i4 | sp\|Q6INL2\|KLH30_XENLA | 41.1 | 73 | 9.09E-06 | 52.4 | Kelch-like protein 30 |  |  |
| 83 |  | TRINITY_DN1222_c0_g1_i1 | sp\|P0C5Q0\|YL54F_YEAST | 85.2 | 27 | 3.67E-08 | 50.1 | Putative uncharacterized protein YLR154W-F |  |  |
|  |  |  |  |  |  |  |  |  |  |  |
|  | **Min** |  |  | **22.6** | **17** | **2.E-208** | **41.6** |  |  |  |
|  | **Max** |  |  | **94.1** | **946** | **5.E-04** | **596** |  |  |  |
|  | **Average** |  |  | **49.28** | **227.48** | **2.E-05** | **168.87** |  |  |  |
|  | **SD** |  |  | **16.42** | **190.86** | **8.E-05** | **127.80** |  |  |  |
