# Supplementary material for: Gene expression profiles of Japanese precious coral Corallium japonicum during gametogenesis
Source: PeerJ. 2024 Apr 16;12:e17182. doi: 10.7717/peerj.17182 (PMC11027906; doi:10.7717/peerj.17182)
Supplement: Supplemental Information 10 [file peerj-12-17182-s010.docx]

**Supplemental Table 4B**. Results of the similarity search for analogous protein sequences (blastp) for male de novo assembled transcripts, and protein annotation (i.e., Molecular function and biological process) from Uniprot database. Data only shows the most significant hits (<0.001).

| **No.** | **Category** | **qaccver** | **saccver** | **pident** | **length** | **evalue** | **bitscore** | **Protein Name** | **Molecular Function** | **Biological Process** |
| --- | --- | --- | --- | --- | --- | --- | --- | --- | --- | --- |
| 1 | Genetic Processes | TRINITY_DN124735_c0_g1_i1 | sp\|Q6W8W5\|POL1_ARMVN | 28.8 | 191 | 4.00E-06 | 51.2 | RNA1 polyprotein | ATP binding, cysteine-type endopeptidase activity, RNA binding, RNA helicase activity, RNA-dependent RNA polymerase activity | DNA-templated transcription, DNA-templated transcription, viral RNA genome replication |
| 2 |  | TRINITY_DN139898_c0_g1_i1 | sp\|P27406\|CAPSD_FCVF9 | 36.1 | 72 | 1.38E-09 | 57.8 | Capsid protein | host cell cytoplasm, T=3 icosahedral viral capsid |  |
| 3 |  | TRINITY_DN21259_c0_g1_i21 | sp\|P34878\|MTSB_LACLC | 30 | 387 | 4.66E-32 | 130 | Type II methyltransferase M2.ScrFI | DNA (cytosine-5-)-methyltransferase activity, DNA Binding | DNA restriction-modification system |
| 4 |  | TRINITY_DN4127_c0_g1_i1 | sp\|P39023\|RL3_HUMAN | 79.4 | 403 | 6.93E-240 | 664 | Large ribosomal subunit protein uL3 | RNA Binding, Structural constituent of ribosome |  |
| 5 |  | TRINITY_DN23049_c0_g1_i4 | sp\|Q99ME9\|GTPB4_MOUSE | 66.3 | 647 | 4.35E-279 | 789 | GTP-binding protein 4 | GTP binding, GTPase activity, Preribosome binding | RNA binding |
| 6 |  | TRINITY_DN48083_c0_g1_i3 | sp\|P84227\|H32_BOVIN | 100 | 100 | 2.69E-61 | 193 | Histone H3.2 | DNA Binding, Protein Heterodimerization activity, Structural constituent of chromatin |  |
| 7 |  | TRINITY_DN3795_c0_g1_i1 | sp\|P32429\|RL7A_CHICK | 78.6 | 266 | 8.28E-143 | 405 | Large ribosomal subunit protein eL8 | RNA Binding | Maturation of LSU-rRNA |
| 8 |  | TRINITY_DN3936_c0_g2_i1 | sp\|Q90596\|MAFK_CHICK | 38 | 108 | 1.06E-09 | 60.1 | Transcription factor MafK | DNA-binding transcription factor activity, DNA-binding transcription factor activity, RNA polymerase II-specific, RNA polymerase II cis-regulatory region sequence-specific DNA binding, sequence-specific DNA binding, transcription cis-regulatory region binding | regulation of transcription by RNA polymerase II |
| 9 |  | TRINITY_DN4516_c0_g2_i4 | sp\|P18395\|CSDE1_RAT | 48.3 | 805 | 1.29E-235 | 711 | Cold shock domain-containing protein E1 | RNA stem-loop binding | stress granule assembly, nuclear-transcribed mRNA catabolic process, no-go decay |
| 10 |  | TRINITY_DN1285_c3_g1_i2 | sp\|Q7LHG5\|YI31B_YEAST | 29 | 1021 | 5.20E-106 | 377 | Transposon Ty3-I Gag-Pol polyprotein | ATP Binding, DNA Binding | DNA integration, DNA recombination, proteolysis, retrotransposition |
| 11 |  | TRINITY_DN1216_c0_g1_i1 | sp\|C0HL66\|H33A_DROME | 100 | 136 | 1.66E-82 | 254 | Histone H3.3A | DNA Binding, Protein Heterodimerization activity, Structural constituent of chromatin | Nucleosome assembly |
| 12 |  | TRINITY_DN30526_c0_g2_i1 | sp\|Q3T057\|RL23_BOVIN | 92.1 | 139 | 1.38E-85 | 251 | Large ribosomal subunit protein uL14 | Structural constituent of ribosome, transcription coactivator binding, ubiquitin protein ligase binding | regulation of G1 to G0 transition, translation |
| 13 |  | TRINITY_DN1818_c0_g1_i3 | sp\|P58375\|RL30_SPOFR | 82.1 | 112 | 2.67E-59 | 182 | Large ribosomal subunit protein eL30 | structural constituent of ribosome |  |
| 14 |  | TRINITY_DN2586_c0_g1_i1 | sp\|O01802\|RL7_CAEEL | 62.9 | 240 | 3.14E-105 | 310 | Large ribosomal subunit protein uL30 | structural constituent of ribosome | maturation of LSU-rRNA from tricistronic rRNA transcript (SSU-rRNA, 5.8S rRNA, LSU-rRNA) |
| 15 |  | TRINITY_DN6204_c0_g1_i1 | sp\|P29694\|EF1G_RABIT | 59.2 | 439 | 1.04E-175 | 504 | Elongation factor 1-gamma | translation elongation factor activitySource:UniProtKB-KW | protein biosynthesis |
| 16 |  | TRINITY_DN20359_c0_g1_i3 | sp\|Q9NR30\|DDX21_HUMAN | 52.8 | 585 | 4.22E-198 | 584 | Nucleolar RNA helicase 2 | RNA Binding | Transcription, Immunity |
| 17 |  | TRINITY_DN2864_c0_g1_i3 | sp\|P22626\|ROA2_HUMAN | 61.3 | 204 | 5.87E-80 | 263 | Heterogeneous nuclear ribonucleoproteins A2/B1 | RNA Binding | mRNA processing, mRNA splicing, mRNA transport |
| 18 |  | TRINITY_DN798_c0_g1_i3 | sp\|P49154\|RS2_URECA | 89 | 236 | 1.11E-146 | 421 | Small ribosomal subunit protein uS5 | RNA Binding, Structural constituent of ribosome | Translation |
| 19 |  | TRINITY_DN3323_c0_g1_i1 | sp\|P46222\|RL11_DROME | 80.9 | 183 | 3.05E-104 | 301 | Large ribosomal subunit protein uL5 | RNA Binding, rRNA binding, structural constituent of ribosome | Cytoplasmic translation |
| 20 |  | TRINITY_DN6363_c0_g1_i1 | sp\|Q90705\|EF2_CHICK | 76.3 | 59 | 2.07E-24 | 98.6 | Elongation factor 2 | GTP binding, GTPase activity, Ribosome binding, Translation elongation factor activity | Translation elongation |
| 21 |  | TRINITY_DN6351_c2_g1_i1 | sp\|P36241\|RL19_DROME | 79.2 | 197 | 1.01E-97 | 286 | Large ribosomal subunit protein eL19 | RNA Binding, Structural constituent of ribosome | Cytoplasmic translation |
| 22 |  | TRINITY_DN31908_c0_g1_i1 | sp\|Q9IA76\|RL31_PAROL | 76.6 | 124 | 2.35E-64 | 195 | Large ribosomal subunit protein eL31 | Structural constituent of ribosome | Translation |
| 23 |  | TRINITY_DN13918_c0_g1_i2 | sp\|O01725\|RLA2_BRAFL | 75 | 68 | 7.46E-27 | 102 | Large ribosomal subunit protein P2 | Structural constituent of ribosome | Cytoplasmic translation elongation |
| 24 |  | TRINITY_DN2733_c0_g1_i5 | sp\|Q56FL2\|RS29_LYSTE | 82.1 | 56 | 1.33E-32 | 112 | Small ribosomal subunit protein uS14 | Structural constituent of ribosome, Zinc ion binding | Cytoplasmic translation |
| 25 |  | TRINITY_DN2760_c0_g1_i1 | sp\|P17844\|DDX5_HUMAN | 67 | 533 | 5.59E-242 | 691 | Probable ATP-dependent RNA helicase DDX5 | RNA Binding | mRNA processing, mRNA splicing, transcription, regulation of osteoblast differentiation, regulation of skeletal muscle cell differentiation, |
| 26 |  | TRINITY_DN2388_c0_g1_i8 | sp\|P32192\|EF1D_ARTSA | 52 | 244 | 8.51E-63 | 202 | Elongation factor 1-delta | translation elongation factor activity |  |
| 27 |  | TRINITY_DN2387_c0_g1_i1 | sp\|P62846\|RS15_CHICK | 85.2 | 142 | 8.67E-83 | 244 | Small ribosomal subunit protein uS19 | RNA binding, structural constituent of ribosome | ribosomal small subunit assembly, translation |
| 28 |  | TRINITY_DN2360_c0_g1_i1 | sp\|Q9VTP4\|R10AB_DROME | 78.6 | 215 | 1.72E-119 | 342 | Large ribosomal subunit protein uL1 | RNA Binding, structural constituent of ribosome | cytoplasmic translation, maturation of LSU- rRNA |
| 29 |  | TRINITY_DN1088_c0_g1_i2 | sp\|Q6F482\|RL39_PLUXY | 86.3 | 51 | 5.16E-25 | 92.8 | Large ribosomal subunit protein eL39 | structural constituent of ribosome | Translation |
| 30 |  | TRINITY_DN29156_c0_g1_i1 | sp\|O18477\|TCTP_LUMRU | 42.1 | 171 | 9.28E-36 | 131 | Translationally-controlled tumor protein homolog |  |  |
| 31 |  | TRINITY_DN14761_c0_g1_i2 | sp\|P62924\|IF5A_SPOEX | 60.8 | 153 | 7.58E-58 | 194 | Eukaryotic translation initiation factor 5A | ribosome binding, RNA binding, translation elongation factor activity | positive regulation of translational elongation and termination |
| 32 |  | TRINITY_DN6482_c0_g1_i1 | sp\|P49165\|RL4_URECA | 75.6 | 386 | 1.80E-197 | 566 | Large ribosomal subunit protein uL4 | structural constituent of ribosome | Translation |
| 33 |  | TRINITY_DN3189_c0_g1_i2 | sp\|Q9BMX5\|RS6_APLCA | 79.8 | 247 | 1.56E-130 | 375 | Small ribosomal subunit protein eS6 | structural constituent of ribosome | Translation |
| 34 |  | TRINITY_DN10367_c0_g1_i1 | sp\|Q94613\|RS19_MYAAR | 68.3 | 139 | 2.49E-67 | 207 | Small ribosomal subunit protein eS19 | structural constituent of ribosome | Translation |
| 35 |  | TRINITY_DN10358_c6_g1_i1 | sp\|P18101\|RL40_DROME | 94.5 | 128 | 4.05E-84 | 247 | Ubiquitin-ribosomal protein eL40 fusion protein | ubiquitin protein ligase binding, structural constituent of ribosome | cytoplasmic translation, proetin ubiquitination, protein modification process |
| 36 |  | TRINITY_DN4402_c0_g1_i1 | sp\|A6QLG5\|RS9_BOVIN | 92.5 | 174 | 3.97E-112 | 323 | Small ribosomal subunit protein uS4 | rRNA binding, structural constituent of ribosome, translation regulator activity | positive regulation of cell population proliferation, ribosomal small subunit biogenesis, translation |
| 37 |  | TRINITY_DN3677_c0_g1_i1 | sp\|Q90YU9\|RL18A_ICTPU | 70.5 | 176 | 2.18E-88 | 261 | Large ribosomal subunit protein eL20 | structural constituent of ribosome | Translation |
| 38 |  | TRINITY_DN6165_c0_g1_i10 | sp\|Q1RLK6\|B3GN4_MOUSE | 29.5 | 207 | 6.94E-17 | 85.9 | N-acetyllactosaminide beta-1,3-N-acetylglucosaminyltransferase 4 | beta-galactosyl-N-acetylglucosaminylgalactosylglucosyl-ceramide beta-1,3-acetylglucosaminyltransferase activity | poly-N-acetyllactosamine biosynthetic process |
| 39 |  | TRINITY_DN1658_c1_g1_i1 | sp\|O96647\|RL10_BOMMA | 81.3 | 209 | 1.54E-122 | 350 | Large ribosomal subunit protein uL16 | structural constituent of ribosome | Translation |
| 40 |  | TRINITY_DN2824_c0_g1_i1 | sp\|Q5XTY7\|RL17_FELCA | 76.1 | 184 | 1.05E-93 | 275 | Large ribosomal subunit protein uL22 | structural constituent of ribosome | Translation |
| 41 |  | TRINITY_DN2850_c0_g1_i1 | sp\|P61356\|RL27_BOVIN | 77.9 | 136 | 7.64E-71 | 214 | Large ribosomal subunit protein eL27 | structural constituent of ribosome | Translation |
| 42 |  | TRINITY_DN36050_c0_g1_i1 | sp\|Q90YV2\|RL15_ICTPU | 78.9 | 204 | 1.33E-112 | 324 | Large ribosomal subunit protein eL15 | structural constituent of ribosome | Translation |
| 43 |  | TRINITY_DN417291_c0_g1_i1 | sp\|Q76N24\|RS4X_CHLAE | 77.2 | 259 | 1.42E-155 | 438 | Small ribosomal subunit protein eS4 | structural constituent of ribosome | Translation |
| 44 | Metabolism and Transport | TRINITY_DN32395_c0_g1_i1 | sp\|Q91X17\|UROM_MOUSE | 56.4 | 94 | 3.76E-31 | 118 | Uromodulin | calcium ion binding, IgG binding | Immunity |
| 45 |  | TRINITY_DN861_c0_g1_i1 | sp\|Q9EQZ5\|PTGR1_CAVPO | 50.4 | 256 | 2.63E-81 | 254 | Prostaglandin reductase 1 | 13-lipoxin reductase activity, 13-prostaglandin reductase activity, 15-oxoprostaglandin 13-oxidase activity, 2-alkenal reductase (NADP+) activity, leukotriene B4 12-hydroxy dehydrogenase activity | Leukotriene B4 metabolic process, lipoxin A4 metabolic process, prostaglandin metabolic process |
| 46 |  | TRINITY_DN27750_c1_g1_i1 | sp\|Q8R4P9\|MRP7_MOUSE | 54.9 | 71 | 2.09E-21 | 95.5 | ATP-binding cassette sub-family C member 10 | ATPase-coupled transmembrane transporter activity, ATP hydrolysis activity, ATP binding, ABC-type xenobiotic transporter activity, ABC-type transporter activity, ABC-type glutathione S-conjugate transporter activity | leukotriene metabolic process, leukotriene transport, lipid transport, transmembrane transport |
| 47 |  | TRINITY_DN15531_c0_g2_i4 | sp\|Q60HD5\|PPOX_MACFA | 39.1 | 220 | 2.09E-42 | 156 | Protoporphyrinogen oxidase | oxygen-dependent protoporphyrinogen oxidase activity | heme biosynthetic process, porphyrin-containing compound biosynthetic process, protoporphyrinogen IX biosynthetic process |
| 48 |  | TRINITY_DN10102_c0_g2_i3 | sp\|Q7TPN3\|PIGV_MOUSE | 33.3 | 93 | 1.41E-04 | 49.7 | GPI mannosyltransferase 2 | glycolipid mannosyltransferase activity |  |
| 49 |  | TRINITY_DN27219_c0_g1_i14 | sp\|Q8AWF2\|NACA_ORENI | 77.6 | 156 | 8.42E-69 | 214 | Nascent polypeptide-associated complex subunit alpha |  | Protein transport |
| 50 |  | TRINITY_DN14038_c0_g2_i6 | sp\|Q5HKG6\|BUTA_STAEQ | 31.1 | 209 | 1.18E-20 | 94 | Diacetyl reductase [(S)-acetoin forming] | diacetyl reductase ((S)-acetoin forming) activity | acetoin catabolic process |
| 51 |  | TRINITY_DN2525_c4_g1_i1 | sp\|P06732\|KCRM_HUMAN | 61.1 | 126 | 5.95E-49 | 166 | Creatine kinase M-type | ATP Binding, Creatinine kinase activity | phosphocreatine biosynthetic process, phosphorylation |
| 52 |  | TRINITY_DN4371_c0_g3_i3 | sp\|P24507\|SY63_DIPOM | 30.9 | 288 | 5.70E-28 | 122 | Synaptotagmin-C | metal ion binding |  |
| 53 |  | TRINITY_DN6202_c0_g1_i5 | sp\|A1KZ92\|PXDNL_HUMAN | 46.4 | 69 | 1.38E-13 | 72.4 | Probable oxidoreductase PXDNL | endonuclease activity, metal ion binding peroxidase activity | hydrogen peroxide catabolic process, response to oxidative stress |
| 54 |  | TRINITY_DN30251_c1_g1_i1 | sp\|P31153\|METK2_HUMAN | 78.8 | 378 | 5.68E-220 | 628 | S-adenosylmethionine synthase isoform type-2 | ATP Binding, Metal ion binding, methionine adenosyltransferase activity | one-carbon metabolic process, protein heterooligomerization, protein hexamerization, S-adenosylmethionine biosynthetic process |
| 55 |  | TRINITY_DN411597_c0_g1_i1 | sp\|Q9IJX4\|POLN_CRPVC | 28.1 | 701 | 9.36E-63 | 243 | Replicase polyprotein | RNA Binding | proteolysis |
| 56 | Cell Structure and Dynamics | TRINITY_DN4631_c0_g1_i6 | sp\|P18294\|KCRF_STRPU | 62.1 | 235 | 2.80E-88 | 296 | Creatine kinase, flagellar | ATP Binding, Creatinine kinase activity | Cell projection organization, phosphocreatinine biosynthetic process, Phosphorylation |
| 57 |  | TRINITY_DN38495_c0_g1_i2 | sp\|A6H584\|CO6A5_MOUSE | 43.8 | 48 | 3.21E-06 | 46.6 | Collagen alpha-5(VI) chain |  | cell adhesion |
| 58 |  | TRINITY_DN6304_c1_g1_i5 | sp\|O88572\|LRP6_MOUSE | 44.1 | 390 | 6.84E-80 | 275 | Low-density lipoprotein receptor-related protein 6 | signaling receptor protein | cell-to-cell adhesion, embyronic development, |
| 59 |  | TRINITY_DN7257_c0_g1_i15 | sp\|A6H584\|CO6A5_MOUSE | 42.2 | 45 | 5.36E-04 | 42.4 | Collagen alpha-5(VI) chain |  | cell adhesion |
| 60 |  | TRINITY_DN23844_c0_g1_i1 | sp\|P20065\|TYB4_MOUSE | 81.4 | 43 | 1.51E-13 | 68.6 | Thymosin beta-4 | actin monomer binding, enzyme binding | actin filament organization, regulation of cell differentaition and migration |
| 61 |  | TRINITY_DN19_c3_g1_i1 | sp\|P02553\|TBA_LYTPI | 99 | 99 | 3.46E-65 | 199 | Tubulin alpha chain | GTP binding, hydrolase activity, structural constituent of cytoskeleton | Microtubule-based process |
| 62 |  | TRINITY_DN15857_c1_g1_i5 | sp\|P02553\|TBA_LYTPI | 99.2 | 118 | 3.00E-84 | 245 | Tubulin alpha chain | structural constituent of cytoskeleton, GTP binding, hydrolase activity | Mircotubule-based process |
| 63 |  | TRINITY_DN6011_c0_g1_i4 | sp\|P11833\|TBB_PARLI | 95.7 | 447 | 0 | 869 | Tubulin beta chain | GTP binding, STPase activity, metal ion binding, structural constituent of cytoskeleton | Microtubule-based process |
| 64 | Cell Cycle and Division | TRINITY_DN14112_c0_g1_i3 | sp\|P26686\|SRR55_DROME | 69.7 | 33 | 1.62E-07 | 53.5 | Serine-arginine protein 55 | mRNA binding | defense response to virus, mitotic cell cycle, mitotic G1/S transition checkpoint signaling, mRNA splicing, via spliceosome, regulation of gene expression, RNA splicing |
| 65 |  | TRINITY_DN21697_c5_g1_i1 | sp\|Q14191\|WRN_HUMAN | 31.2 | 154 | 4.04E-16 | 82.8 | Bifunctional 3'-5' exonuclease/ATP-dependent helicase WRN | DNA binding | DNA damage response |
| 66 |  | TRINITY_DN6639_c0_g3_i1 | sp\|Q09811\|HUS2_SCHPO | 27.3 | 439 | 1.28E-35 | 147 | ATP-dependent DNA helicase hus2/rqh1 | DNA binding | DNA damage response |
| 67 |  | TRINITY_DN6620_c1_g1_i7 | sp\|D3YXG0\|HMCN1_MOUSE | 24 | 288 | 2.14E-16 | 87 | Hemicentin-1 | Calcium ion binding | cell cycle, cell division, heterophilic cell-cell adhesion via plasma membrane cell adhesion molecules, homophilic cell adhesion via plasma membrane adhesion molecules |
| 68 | Growth and Development | TRINITY_DN13284_c0_g1_i2 | sp\|Q9BMN8\|LAR_CAEEL | 25.5 | 267 | 3.58E-05 | 48.5 | Tyrosine-protein phosphatase Lar-like | protein tyrosine phosphatase activity, transmembrane receptor protein tyrosine phosphatase activity | embryonic body morphogenesis, epidermis development, gastrulation, motor neuron axn guidance, nervous system development, neuroblast/neuron migration, protein dephosphorylation, synapse assembly |
| 69 |  | TRINITY_DN14927_c0_g1_i17 | sp\|F1QB54\|PABPA_DANRE | 67.1 | 668 | 5.97E-274 | 796 | Polyadenylate-binding protein 1A | mRNA 3'-UTR binding, poly(A) binding, poly(U) RNA binding | heart development, mRNA processing, regulation of translation |
| 70 | Cellular Signaling and Response | TRINITY_DN2642_c0_g1_i1 | sp\|Q56JV9\|RS3A_BOVIN | 80.7 | 264 | 5.37E-151 | 437 | Small ribosomal subunit protein eS1 | mRNA 5'-UTR binding, structural constituent of ribosome | cell differentiation, negative regulation of apoptotic process, ribosomal small subunit biogenesis, translation |
| 71 |  | TRINITY_DN484_c0_g1_i1 | sp\|P08108\|HSP70_ONCMY | 87.3 | 655 | 0 | 1087 | Heat shock cognate 70 kDa protein | ATP binding, ATP-dependent protein folding chaperone | response to arsenite ion, cadmium ion, copper ion, heat |
| 72 |  | TRINITY_DN1913_c0_g1_i51 | sp\|Q5XXA9\|PSIP1_CHICK | 36.1 | 108 | 7.81E-07 | 53.5 | Lens epithelium-derived growth factor | DNA-binding transcription factor binding, supercoiled DNA binding, transcription coactivator activity | positive regulation of transcription by RNA polymerase II, response to heat, response to oxidative stress |
|  |  |  |  |  |  |  |  |  |  |  |
|  |  | **Min** |  | **24** | **33** | **0.E+00** | **42.4** |  |  |  |
|  |  | **Max** |  | **100** | **1021** | **5.E-04** | **1087** |  |  |  |
|  |  | **Average** |  | **63.65** | **245.81** | **1.E-05** | **276.54** |  |  |  |
|  |  | **StDEv** |  | **22.53** | **197.58** | **7.E-05** | **224.07** |  |  |  |
