# Supplementary material for: Gene expression profiles of Japanese precious coral Corallium japonicum during gametogenesis
Source: PeerJ. 2024 Apr 16;12:e17182. doi: 10.7717/peerj.17182 (PMC11027906; doi:10.7717/peerj.17182)
Supplement: Supplemental Information 11 [file peerj-12-17182-s011.docx]

**Supplemental Table 5.** List of genes of interest that were upregulated only in female *Corallium japonicum*.

| **Biological Process Terms** | **Contig ID** | **qseqid\|sseqid** | **Annotation** | **E value** |
| --- | --- | --- | --- | --- |
| Reproduction | Contig_32496 | gi\|9963975\|gb\|AF248497.1\|AF248497 | Vg1 mRNA, complete cds | 4.92E-04 |
| Cell cycle | Contig_7184 | gi\|10336\|emb\|X15485.1\| | cyclin B | 3.10E-27 |
|  | Contig_11661 | gi\|1317983795\|ref\|XM_012420498.2\| | cyclin-dependent kinases regulatory subunit | 1.17E-15 |
|  | Contig_25058 | gi\|1270079407\|ref\|XM_022947167.1\| | cyclin-J-like | 1.03E-12 |
|  | Contig_32685 | gi\|585681596\|ref\|XM_002737469.2\| | cyclin-dependent kinase 1-like | 1.66E-99 |
|  | Contig_35869 | gi\|1042365036\|ref\|XM_017471111.1\| | G2/mitotic-specific cyclin-B3-like | 3.67E-26 |
| Cell division | Contig_31564 | gi\|1032756281\|ref\|XM_008215079.2\| | fizzy (fzy) | 3.41E-21 |
|  | Contig_32685 | gi\|11034745\|dbj\|AB050461.1\| | serine/threonine kinase cdc2 | 7.07E-60 |
|  | Contig_41918 | gi\|1176078661\|ref\|XM_020753731.1\| | follicle-stimulating hormone receptor-like | 7.E-13 |
| Cell population proliferation | Contig_3066 | gi\|1211337955\|ref\|XM_021593677.1\| | BMP-binding endothelial regulator protein-like | 8.E-32 |
|  | Contig_9007 | gi\|339247122\|ref\|XM_003375147.1\| | RNA-binding protein 24 (Tsp_04382) | 4.E-06 |
| Macromolecule metabolic process | Contig_17293 | gi\|929088025\|ref\|XM_014199181.1\| | cyclin A2 (ccna2) | 8.E-10 |
|  | Contig_17293 | gi\|1049362535\|ref\|XM_008637075.2\| | cyclin B2 (CCNB2) | 2.E-04 |
|  | Contig_36030 | gi\|723137510\|ref\|XM_010287192.1\| | stem-loop binding protein (SLBP) | 5.E-04 |
| Nitrogen compound metabolic process | Contig_17293 | gi\|1005442668\|ref\|XM_015903186.1\| | G2/mitotic-specific cyclin-B-like | 1.83E-17 |
|  | Contig_17805 | gi\|242019413\|ref\|XM_002430111.1\| | G1/S-specific cyclin-C | 9.59E-39 |
|  | Contig_36030 | gi\|1005477917\|ref\|XM_015920233.1\| | histone RNA hairpin-binding protein-like | 2.24E-08 |
|  | Contig_6045 | gi\|1005460150\|ref\|XM_015911666.1\| | DNA excision repair protein ERCC-6-like | 2.13E-62 |
| Organelle organization | Contig_6045 | gi\|1005478005\|ref\|XM_015920269.1\| | histone H2B, gonadal-like | 2.73E-81 |
|  | Contig_39917 | gi\|340385510\|ref\|XM_003391205.1\| | histone H2A | 1.05E-71 |
