# Supplementary material for: Gene expression profiles of Japanese precious coral Corallium japonicum during gametogenesis
Source: PeerJ. 2024 Apr 16;12:e17182. doi: 10.7717/peerj.17182 (PMC11027906; doi:10.7717/peerj.17182)
Supplement: Supplemental Information 12 [file peerj-12-17182-s012.docx]

**Supplemental Table 6.** List of genes that enriched cell adhesion and their corresponding biological function. Genes that are not included do not have matched annotation.

| **Contig ID** | **qseqid\|sseqid** | **Annotation** | **E value** |
| --- | --- | --- | --- |
| Contig_28006 | gi\|1005473645\|ref\|XR_001566203.1\| | focal adhesion kinase 1-like | 5.92E-15 |
|  | gi\|1176119311\|ref\|XM_020775242.1\| | GTP-binding protein ypt1-like | 5.19E-22 |
|  | gi\|1176064474\|ref\|XM_020746755.1\| | phosphatidylinositol-glycan-specific phospholipase D-like | 3.07E-12 |
|  | gi\|1176096592\|ref\|XM_020763251.1\| | vascular endothelial growth factor A-like | 5.19E-22 |
| Contig_28440 | gi\|1176076889\|ref\|XM_020752810.1\| | sushi domain-containing protein 2-like | 1.72E-21 |
|  | gi\|1249003295\|ref\|XM_022675471.1\| | sushi, nidogen and EGF-like domain-containing protein 1 | 1.72E-21 |
