# Supplementary material for: Gene expression profiles of Japanese precious coral Corallium japonicum during gametogenesis
Source: PeerJ. 2024 Apr 16;12:e17182. doi: 10.7717/peerj.17182 (PMC11027906; doi:10.7717/peerj.17182)
Supplement: Supplemental Information 13 [file peerj-12-17182-s013.docx]

**Supplemental Table 7.** Raw data of the gonad diameters measured using Image J software.

| **Sex** | **Sample ID** | **Image No.** | **Gonad No.** | **Diameter (μm)** | **Average (μm)** | **Standard Deviation** |
| --- | --- | --- | --- | --- | --- | --- |
| Female | PC01 | No. 1 | 1 | 72.55 | **93.64** | **23.96** |
|  | PC01 | No. 1 | 2 | 69.15 |  |  |
|  | PC01 | No. 1 | 3 | 92 |  |  |
|  | PC01 | No. 2 | 4 | 118.55 |  |  |
|  | PC01 | No. 2 | 5 | 132.8 |  |  |
|  | PC01 | No. 3 | 6 | 92.25 |  |  |
|  | PC01 | No. 3 | 7 | 78.15 |  |  |
| Female | PC02 | No. 1 | 8 | 147.3 | **157.34** | **31.88** |
|  | PC02 | No. 1 | 9 | 119.95 |  |  |
|  | PC02 | No. 1 | 10 | 148.5 |  |  |
|  | PC02 | No. 1 | 11 | 113.8 |  |  |
|  | PC02 | No. 2 | 12 | 203.55 |  |  |
|  | PC02 | No. 2 | 13 | 185.1 |  |  |
|  | PC02 | No. 3 | 14 | 184.4 |  |  |
|  | PC02 | No. 3 | 15 | 156.1 |  |  |
| Male | PC03 | No. 1 | 1 | 69.9 | **56.11** | **10.72** |
|  | PC03 | No. 2 | 2 | 57.9 |  |  |
|  | PC03 | No. 2 | 3 | 61.45 |  |  |
|  | PC03 | No. 3 | 4 | 48.7 |  |  |
|  | PC03 | No. 3 | 5 | 42.6 |  |  |
| Male | PC04 | No. 1 | 6 | 42.4 | **46.8** | **6.34** |
|  | PC04 | No. 1 | 7 | 42.45 |  |  |
|  | PC04 | No. 2 | 8 | 39.8 |  |  |
|  | PC04 | No. 2 | 9 | 47.65 |  |  |
|  | PC04 | No. 3 | 10 | 55.25 |  |  |
|  | PC04 | No. 3 | 11 | 53.25 |  |  |
| **Average** | | | | **95.21** |  | |
| **Standard Deviation** | | | | **50.52** |  | |
